# Immunotherapy in Gynecologic Cancers: Current Evidence, Biomarker-Driven Practice, and Future Directions

**DOI:** 10.3390/cancers18172771

**Published:** 2026-08-26

**Authors:** Chen Yang, Yuxiao Wu, Junjun Yang, Yang Xiang

**Affiliations:** National Clinical Research Center for Women’s Health and Obstetric and Gynecologic Diseases, Department of Obstetrics and Gynecology, Peking Union Medical College Hospital, Chinese Academy of Medical Sciences & Peking Union Medical College, Beijing 100730, China; yangchen46@pumch.cn (C.Y.); s2025001139@student.pumc.edu.cn (Y.W.); yangjunjun@pumch.cn (J.Y.)

**Keywords:** gynecologic cancer, immunotherapy, immune checkpoint inhibitor, cervical cancer, endometrial cancer, ovarian cancer, gestational trophoblastic neoplasia, biomarker

## Abstract

Immune checkpoint blockade has become an important component of treatment for several gynecologic malignancies, but its clinical value varies substantially by tumor lineage, molecular subtype, disease setting, and treatment backbone. This narrative review summarizes the biological rationale, clinical evidence, predictive biomarkers, combination strategies, toxicity considerations, and future directions of immunotherapy across gynecologic cancers. In cervical cancer, checkpoint blockade has evolved from later-line recurrent disease to first-line systemic therapy and, more recently, to curative-intent chemoradiotherapy combinations. In endometrial cancer, mismatch repair-deficient/microsatellite instability-high (dMMR/MSI-H) tumors show the clearest sensitivity to programmed cell death protein 1 (PD-1) blockade, whereas mismatch repair-proficient/microsatellite stable (pMMR/MSS) disease generally requires combination strategies. In ovarian cancer, broad unselected checkpoint inhibitor strategies have generally failed; ENGOT-ov65/KEYNOTE-B96 demonstrated statistically significant progression-free survival (PFS) and overall survival (OS) improvement with pembrolizumab plus weekly paclitaxel, with or without bevacizumab, in a defined platinum-resistant population, although the absolute median PFS gain in PD-L1 combined positive score (CPS) ≥1 disease was modest. Gestational trophoblastic neoplasia (GTN) offers a biologically distinctive setting because of trophoblastic immune tolerance, whereas selected rare gynecologic malignancies may be considered for immunotherapy on the basis of HPV association, tumor-agnostic biomarkers, or signals observed in clear-cell cohorts. Accordingly, the clinical value of immunotherapy should be interpreted according to tumor lineage, disease setting, validated biomarkers, treatment backbone, and the incremental contribution of each treatment component.

## Simple Summary

Immunotherapy has changed the treatment of several gynecologic cancers, but its benefit varies greatly between tumor types and clinical settings. The clearest benefits are seen when treatment is matched to tumor biology, such as mismatch repair deficiency in endometrial cancer or PD-L1 expression in selected cervical and ovarian cancer settings. In ovarian cancer, most unselected immunotherapy strategies have been unsuccessful, although a recent phase III study identified a treatment option for a selected platinum-resistant population with only a modest absolute gain in progression-free survival. Patients with rare tumors, including gestational trophoblastic neoplasia, may also benefit in selected circumstances. This review focuses on how tumor biology, biomarkers, treatment setting, toxicity, and prior therapy should guide the appropriate use and future development of immunotherapy.

## 1. Introduction

Gynecologic cancers comprise a biologically heterogeneous group of malignancies, including cervical, endometrial, ovarian, vulvar, vaginal, and gestational trophoblastic neoplasia (GTN). Together, they account for a major global burden of cancer incidence and mortality among women. Recent global estimates indicate that gynecologic cancers caused more than 1.47 million new cases and approximately 680,000 deaths worldwide in 2022, with cervical, endometrial, and ovarian cancers contributing the largest share [1,2]. These diseases differ markedly in etiology, molecular pathogenesis, natural history, patterns of spread, sensitivity to local therapy, and systemic treatment options. Accordingly, immunotherapy must be interpreted within the distinct biological and clinical context of each gynecologic malignancy rather than as a uniform treatment strategy.

The therapeutic landscape of gynecologic cancers has changed substantially during the past decade. Conventional local and systemic modalities remain essential, but newer treatment backbones—including anti-angiogenic therapy, endocrine therapy, poly(ADP-ribose) polymerase (PARP) inhibitors, human epidermal growth factor receptor 2 (HER2)-directed therapy, antibody-drug conjugates (ADCs), and immune checkpoint inhibitors (ICI)—have reshaped selected disease settings [3,4,5,6,7,8,9,10,11,12,13,14,15,16,17,18,19,20,21,22,23,24,25,26,27]. The magnitude of benefit, however, varies by tumor type, molecular subtype, disease setting, and treatment backbone. Immune checkpoint blockade targets inhibitory pathways that restrain antitumor T-cell activity, but responses remain heterogeneous even among tumors with immune-cell infiltration or checkpoint expression [28,29,30,31,32,33,34,35].

These divergent clinical outcomes reflect distinct tumor immunobiology. HPV-associated tumors provide persistent viral antigens; dMMR/MSI-H and POLE-mutated endometrial cancers generate highly immunogenic mutational landscapes; ovarian cancer is frequently constrained by spatial immune exclusion and suppressive stromal and myeloid programs; and gestational trophoblastic neoplasia combines paternal alloantigenicity with trophoblastic immune tolerance [36,37,38,39,40,41,42,43,44,45,46,47,48,49,50,51,52,53,54,55,56,57,58,59,60,61,62,63,64,65,66,67,68,69,70,71,72,73,74,75,76,77,78].

This review critically evaluates clinical evidence, biomarker-defined treatment selection, combination strategies, toxicity, survivorship, and post-ICI sequencing across gynecologic cancers. Distinct from reviews focused primarily on checkpoint inhibitor efficacy, this review integrates recent practice-changing and negative phase III evidence, disease-specific biomarker interpretation, rare trophoblastic and clear-cell contexts, and the emerging challenge of treatment sequencing after prior immunotherapy exposure. We distinguish established practice from biologically attractive but unproven extrapolation and interpret treatment value according to disease setting, absolute benefit, available alternatives, cumulative toxicity, and long-term survivorship implications. Pivotal and practice-informing clinical studies are summarized in Table 1, whereas supportive early-phase, retrospective, and rare-tumor studies are provided in Supplementary Table S1.

Review approach: This narrative review was informed by searches of PubMed/MEDLINE, ClinicalTrials.gov, major oncology congress reports, and major international guidelines, with no lower date restriction and with searches updated through July 2026. Search concepts combined disease-specific terms for cervical, endometrial, ovarian, vulvar, vaginal, and gestational trophoblastic malignancies with immunotherapy, PD-1, PD-L1, CTLA-4, immune checkpoint inhibitor, biomarker, resistance, and the names of pivotal trials. We prioritized randomized phase III studies, pivotal biomarker-defined phase I/II studies, and high-quality translational studies. Positive, negative, and inconclusive studies were retained when they materially informed clinical practice, biomarker interpretation, treatment sequencing, or biological understanding. Foundational studies were included when necessary to establish key immunobiological concepts or molecular classifications, whereas evidence from small prospective cohorts, retrospective series, or selected case-based reports was considered primarily for rare gynecologic malignancies when higher-level evidence was unavailable. Because this was a narrative rather than a systematic review, we did not perform a formal risk-of-bias assessment or quantitative meta-analysis. For consistency, evidence was interpreted using a pragmatic clinical framework. Strategies were categorized as established/practice-informing when supported by mature randomized evidence or incorporated into contemporary treatment pathways; biomarker- or setting-selected when benefit was confined to a defined molecular, clinical, histologic, or treatment context; investigational/emerging when supported mainly by early-phase or nonrandomized evidence; and negative/inconclusive when randomized evidence failed to demonstrate benefit or when the incremental contribution of immunotherapy could not be established.

## 2. Biological Rationale and Biomarkers

### 2.1. The Cancer-Immunity Cycle and Checkpoint Biology

The heterogeneous activity of immunotherapy across gynecologic cancers can be understood through the cancer-immunity cycle. Durable antitumor immunity requires malignant cells to release recognizable antigens, those antigens to be presented effectively, and effector T cells to reach and kill tumor cells within a permissive microenvironment. Disruption of any of these steps can lead to immune escape, primary resistance, or acquired resistance. The PD-1/PD-L1 pathway is central to many current strategies because it suppresses T-cell receptor signaling, cytokine production, and cytotoxic function in chronically stimulated T cells [28,29]. Antibodies against PD-1 or PD-L1 can restore immune activity in selected patients, but only when the broader immune context remains capable of supporting an antitumor response.

Cancer immunoediting provides a complementary evolutionary framework for understanding immune escape. Through the phases of elimination, equilibrium, and escape, immune pressure can shape tumor clonal composition by eliminating highly immunogenic cells while permitting less immunogenic or more immune-evasive populations to persist and expand. Therapeutic checkpoint blockade may impose additional selective pressure, potentially favoring clones with reduced antigenicity, impaired antigen processing or HLA presentation, altered interferon signaling, or other mechanisms of immune evasion [94,95]. Thus, immunotherapy response is not static but can evolve over time as both tumor-intrinsic and microenvironmental features are reshaped by immune selection.

**Figure 1:**
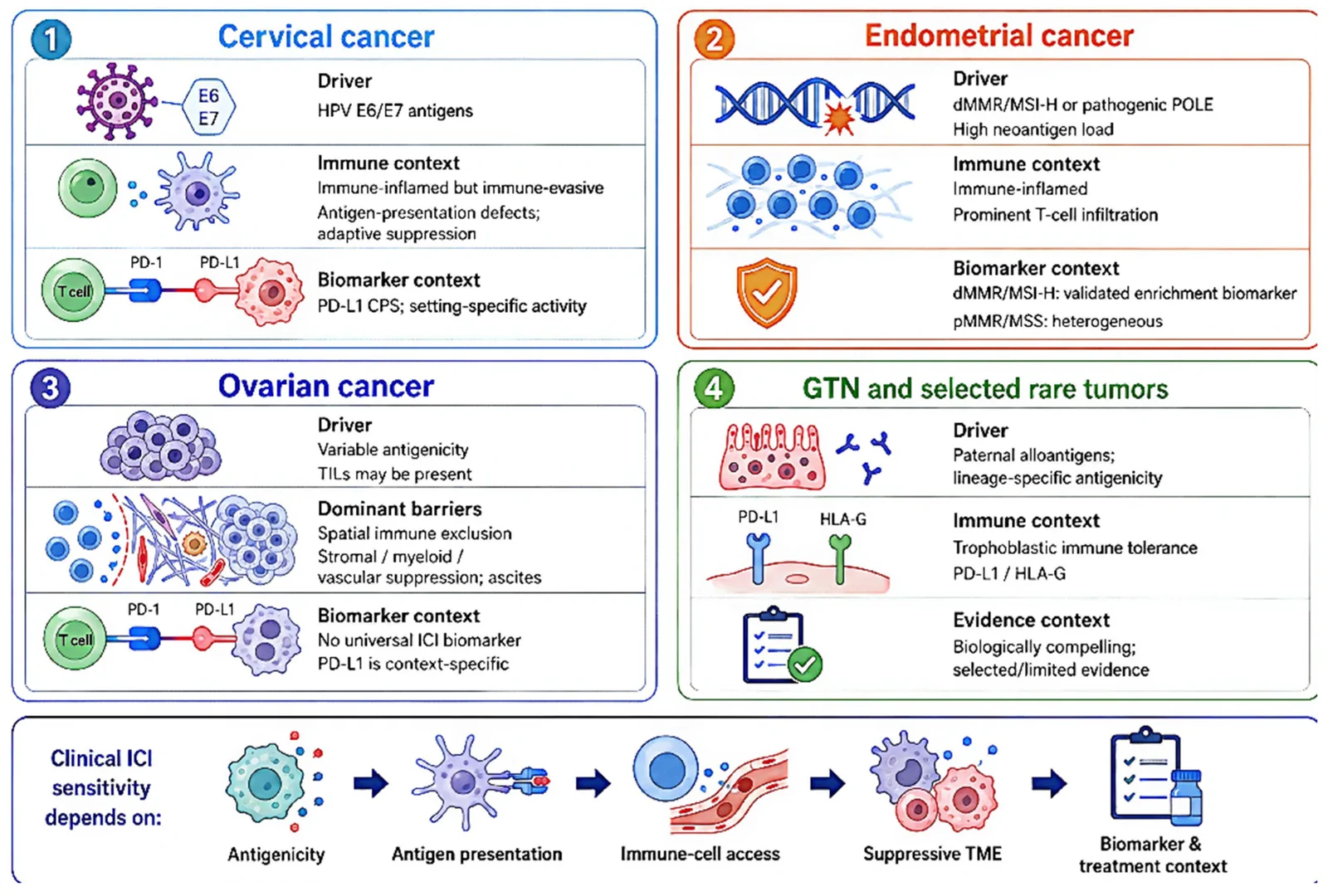
Immunobiological determinants of checkpoint sensitivity across gynecologic cancers. Tumor types differ in antigenicity, antigen-presentation capacity, immune-cell access, suppressive microenvironmental programs, and biomarker performance. Clinical ICI sensitivity reflects the interaction of these biological features with treatment context rather than any single immune characteristic. Abbreviations: CPS, combined positive score; dMMR, mismatch repair-deficient; E6/E7, human papillomavirus early proteins 6 and 7; GTN, gestational trophoblastic neoplasia; HLA-G, human leukocyte antigen G; HPV, human papillomavirus; ICI, immune checkpoint inhibitor; MSI-H, microsatellite instability-high; MSS, microsatellite stable; PD-1, programmed cell death protein 1; PD-L1, programmed death-ligand 1; pMMR, mismatch repair-proficient; POLE, DNA polymerase epsilon; TIL, tumor-infiltrating lymphocyte; TME, tumor microenvironment.

The broader immuno-oncology literature has refined this framework by showing that checkpoint inhibitor response is shaped by the quality of tumor antigens, the integrity of antigen presentation and interferon signaling, host factors such as HLA genotype, and the spatial relationship between immune and malignant cells [30,31,32,33,34,35,94,95,96,97,98]. These concepts are directly relevant to gynecologic cancers. A PD-L1-positive cervical tumor with persistent HPV antigen expression may behave differently from a PD-L1-positive ovarian tumor in which T cells are largely excluded from epithelial tumor nests. Similarly, the immunogenicity of a high tumor mutational burden depends on whether neoantigens can be presented and recognized. Biomarker interpretation therefore requires lineage-specific and context-specific judgment rather than application of a single threshold without disease- and treatment-specific context. Resistance to checkpoint blockade is also shaped by nonmalignant components of the tumor microenvironment. Cancer-associated fibroblasts and extracellular-matrix programs can create physical and chemokine-mediated barriers that restrict effector T-cell access to malignant cells, whereas myeloid-derived suppressor cells and suppressive macrophage populations can impair antigen presentation, inhibit T-cell activation, and reinforce immunosuppressive cytokine and metabolic states. Regulatory T cells further restrain cytotoxic effector function and can maintain local immune tolerance [32,33,34,35,57,59,60,61,62,63,64,65,66,67,68,69,70]. These mechanisms are particularly evident in immune-excluded ovarian cancer but are relevant, to varying degrees, across gynecologic malignancies. Their relative contribution depends on tumor lineage, molecular subtype, disease state, and prior treatment, which may partly explain why the same checkpoint-based combination does not produce equivalent benefit across tumor types. The major immunobiological determinants of checkpoint sensitivity across gynecologic cancers are summarized in Figure 1.

### 2.2. Viral Antigenicity in HPV-Associated Tumors

HPV-associated tumors provide one of the strongest rationales for immunotherapy in gynecologic oncology. Persistent infection with high-risk HPV is the principal driver of most cervical cancers and contributes to subsets of vulvar and vaginal squamous cell carcinomas [42,43,44,45]. Viral E6 and E7 oncoproteins promote malignant transformation by disrupting p53 and RB pathway function and remain constitutively expressed in tumor cells, making them potential targets for immune recognition [36,37,46]. This biology supports checkpoint blockade, therapeutic vaccines, adoptive tumor-infiltrating lymphocyte therapy, and engineered T-cell receptor (TCR) approaches, although the level of clinical evidence differs substantially among these strategies [4,5,6,7,8,9,10,11,79,80,99,100,101,102,103,104,105,106,107].

Viral antigenicity, however, does not guarantee effective immune elimination or universal sensitivity to checkpoint blockade. Persistent E6/E7 expression provides non-self antigens, but successful antitumor immunity additionally requires efficient antigen processing and HLA presentation, recognition by functional effector T cells, and access of those cells to malignant tissue within a permissive microenvironment. HPV-associated tumors may evade immune destruction through impaired antigen-processing or HLA pathways, altered interferon responsiveness, chronic antigen-driven T-cell dysfunction and adaptive checkpoint upregulation, recruitment of regulatory T cells and suppressive myeloid populations, and stromal or vascular barriers that restrict effector-cell access [32,33,34,35,42,43,44,45,46,108]. Thus, persistent viral antigen expression provides a biological rationale for immunotherapy but is not itself a sufficient predictive biomarker of response. Vaccines directed against HPV oncoproteins can generate antigen-specific immunity, but established invasive cancers may require combination strategies because immune priming alone may be insufficient to overcome these tumor-intrinsic and microenvironmental escape mechanisms [46,102,107]. This biology helps explain the modest response rates observed with single-agent PD-1 blockade in recurrent cervical cancer and why greater clinical activity may be observed in appropriately selected combination-treatment settings [4,5,6,7,8,9,10,11,79,80,99,100,101].

### 2.3. Mismatch Repair Deficiency, POLE Mutation, and Endometrial Cancer

Endometrial cancer is the clearest biomarker-driven model for gynecologic immunotherapy. The Cancer Genome Atlas (TCGA) classified endometrial carcinoma into POLE-ultramutated, microsatellite instability (MSI)-hypermutated, copy-number low, and copy-number high subgroups, providing a molecular framework with direct implications for prognosis and treatment selection [38]. dMMR/MSI-H tumors accumulate insertion-deletion mutations and frameshift-derived neoantigens, leading to immune infiltration and adaptive checkpoint activation. Across solid tumors, mismatch repair deficiency predicts response to PD-1 blockade, and in endometrial cancer it identifies the subgroup with the greatest magnitude of benefit from immunotherapy in recurrent and first-line settings [15,16,17,18,19,39,40,52,53,109]. However, dMMR/MSI-H is an enrichment biomarker rather than a deterministic predictor of response. Immunogenicity generated by mismatch-repair deficiency does not ensure effective antitumor immunity when downstream processes such as antigen presentation, interferon signaling, effector-cell trafficking, or the local immune microenvironment remain unfavorable [30,31,32,33,34,35,94,95,110].

POLE-mutated endometrial cancers represent a related but distinct immunogenic subgroup. Pathogenic exonuclease-domain mutations produce an ultramutated phenotype, abundant predicted neoantigens, and prominent immune infiltration [41]. Yet these tumors usually have an excellent prognosis, even when histologic features appear high risk. The more clinically relevant question is whether selected patients can undergo safe treatment de-escalation, an approach that requires validated molecular classification and prospective confirmation. Clinically applicable molecular classifiers, validation studies, and POLE interpretation frameworks have helped translate TCGA categories into routine practice, whereas analyses of adjuvant trial cohorts demonstrate that molecular subtype can modify prognosis and treatment benefit [47,48,49,50,51,55].

### 2.4. Immune Suppression and Spatial Exclusion in Ovarian Cancer

Ovarian cancer illustrates that prognostic immunity and therapeutic responsiveness are not the same. Intratumoral T cells, CD8-positive infiltration, tissue-resident memory phenotypes, and favorable CD8-to-regulatory T-cell balance have been associated with improved outcomes in epithelial ovarian cancer [56,57,58,63,64,65]. These observations established that antitumor immunity is biologically meaningful. Nevertheless, single-agent PD-1 or PD-L1 inhibitors have produced only modest activity, and several earlier large randomized trials failed to establish checkpoint blockade as a standard for unselected epithelial ovarian cancer [83,84,86,87,111,112].

Several mechanisms help explain this discrepancy. High-grade serous ovarian cancer is characterized by near-universal TP53 mutation, extensive copy-number alterations, intratumoral heterogeneity, and frequent homologous recombination deficiency (HRD), but these features do not necessarily create the immune-inflamed phenotype seen in dMMR endometrial cancer. Instead, the ovarian cancer microenvironment often combines suppressive lymphoid and myeloid populations, fibroblast-rich stroma, abnormal vasculature, hypoxia, and metabolic stress [57,59,60,61,62,63,64,65,66,67,68,69,70]. Malignant ascites adds a separate immune compartment that can further impair T-cell function and promote treatment resistance.

Spatial biology is particularly important. Immune cells may be present in ovarian tumors but confined to stromal regions, separated from malignant epithelial nests, or organized into focal aggregates that do not support broad tumor-cell killing. Recent single-cell, spatial, and perturbational studies have begun to link these architectures to malignant cell states, genetic programs, and candidate regulators of immune evasion [59,60,61,66,67,68,69,70]. These spatial and single-cell findings support a shift from adding checkpoint blockade empirically to developing strategies that improve immune-cell access to malignant epithelial nests.

### 2.5. Trophoblastic Immune Tolerance and Rare Tumor Contexts

Gestational trophoblastic neoplasia is immunologically distinctive because it arises from gestational tissue and may contain paternal alloantigens while retaining mechanisms of maternal-fetal tolerance. Trophoblasts express immune regulatory molecules, including PD-L1 and non-classical HLA molecules such as HLA-G, that help protect the fetus from maternal immune rejection. These same mechanisms may be co-opted by trophoblastic tumors [71,72,73,74]. The combination of foreign antigenicity and immune tolerance provides a compelling rationale for PD-1/PD-L1 blockade in chemotherapy-resistant GTN. Rare gynecologic tumors may also represent immunotherapy niches when HPV-associated antigenicity, clear cell histology, or tumor-agnostic biomarkers provide a specific biological rationale [75,76,77,78,105,106,113,114,115,116,117,118].

### 2.6. Clinically Relevant and Emerging Biomarkers

Several biomarkers are already clinically relevant. dMMR/MSI-H is the most robust clinically validated enrichment biomarker in endometrial cancer and has tumor-agnostic relevance for PD-1 blockade [39,40,52,53,109]. PD-L1 CPS is clinically actionable only in defined treatment settings. In cervical cancer, CPS ≥ 1 supports pembrolizumab-containing first-line therapy for persistent, recurrent, or metastatic disease. In ovarian cancer, CPS ≥ 1 identifies patients with platinum-resistant epithelial ovarian, fallopian tube, or primary peritoneal cancer for whom pembrolizumab plus weekly paclitaxel, with or without bevacizumab, has demonstrated benefit after one or two prior systemic regimens [4,5,6,7,85,99]. PD-L1 should not be regarded as a universal predictor of checkpoint inhibitor sensitivity across gynecologic cancers. PD-L1 CPS also has important analytical and biological limitations. PD-L1 expression is spatially heterogeneous within tumors and may differ between primary and metastatic sites, while use of archival tissue may not reflect the immune state of contemporary disease. Expression is also dynamic and can be altered by interferon signaling, chemotherapy, radiotherapy, and other prior treatments. CPS is also sensitive to assay platform, specimen characteristics, and scoring reproducibility, limiting the portability of a single threshold across treatment settings [29,35]. These limitations help explain why PD-L1 performs differently across tumor types and treatment settings and why positivity enriches for benefit in some settings without reliably identifying all responders. TMB-high status represents a potential tumor-agnostic biomarker for checkpoint blockade and, depending on regulatory jurisdiction, may support pembrolizumab use in selected patients with previously treated unresectable or metastatic solid tumors for whom satisfactory alternatives are unavailable [119]. In gynecologic cancers, however, TMB should be regarded as a contextual enrichment biomarker rather than a universal predictor of response. Its clinical relevance depends not only on mutational quantity but also on assay platform and threshold, tumor lineage, neoantigen quality, HLA and antigen-presentation integrity, and whether the tumor microenvironment permits effective immune-cell recognition and access [30,35,96,97,98,119]. Accordingly, TMB-high status should complement rather than substitute for disease-specific biomarkers such as MMR/MSI status, pathogenic POLE mutation, and established molecular classification.

Future biomarker development will likely require composite models rather than single markers. In practice, such models may need to integrate tumor lineage, viral or mutational antigenicity, molecular subtype, antigen-presentation capacity, immune-cell state, vascular or myeloid suppression, spatial architecture, and dynamic markers such as circulating tumor DNA (ctDNA) clearance [30,31,32,33,34,35,47,48,49,50,51,52,53,54,55,62,63,64,65,66,67,68,69,70,94,95,96,97,98,120,121]. Biomarkers may also need to be treatment-specific. VEGF has immunologic effects beyond its central role in angiogenesis. VEGF-driven abnormal tumor vasculature can impair effective lymphocyte trafficking into the tumor microenvironment, while VEGF signaling can interfere with dendritic-cell maturation and T-cell priming and contribute to T-cell dysfunction and suppressive myeloid or regulatory immune programs [122]. Anti-angiogenic therapy may partially normalize tumor vasculature and vascular–immune crosstalk, improve effector-cell access, and alleviate selected immunosuppressive features, thereby providing a biologic rationale for combination with checkpoint blockade. However, this mechanistic complementarity should not be equated with clinical synergy, which remains dependent on tumor lineage, treatment backbone, dose and scheduling, and patient selection. PARP inhibition can activate innate immune signaling through DNA damage and the cyclic GMP-AMP synthase-stimulator of interferon genes (cGAS-STING) pathway [122,123]. The challenge is to convert these mechanisms into clinically validated selection tools rather than assuming that mechanistic plausibility alone predicts patient benefit.

Practical biomarker implementation depends not only on assay validity but also on specimen adequacy, laboratory workflow, turnaround time, and interpretive expertise. MMR immunohistochemistry and PD-L1 CPS testing can generally be performed on routine formalin-fixed, paraffin-embedded tissue and are typically available more rapidly than sequencing-based assays. However, PD-L1 assessment requires adequate viable tumor and immune-cell representation and remains sensitive to antibody clone, platform, specimen, inflammatory context, and scoring method [4,5,6,7,35,99,118]. MMR immunohistochemistry requires appropriate internal controls, whereas MSI can be assessed by PCR- or next-generation sequencing-based approaches; discordant MMR and MSI results warrant careful pathology and molecular review [20,21,47,48,49,52,53,109]. By contrast, POLE and TMB assessment generally requires sufficient tumor content and nucleic-acid quality for sequencing and may entail longer processing times than single-marker immunohistochemistry. Small or extensively necrotic specimens and low-tumor-content samples can limit assay success, whereas archival tissue may not fully represent the current biology of dynamic biomarkers such as PD-L1. POLE interpretation additionally requires distinction of pathogenic exonuclease-domain mutations from variants of uncertain significance because misclassification may lead to inappropriate treatment de-escalation or intensification [50,55]. When assay results, molecular classification, and clinicopathologic features are discordant, repeat or orthogonal testing and expert pathology or molecular review should be considered. Spatial and single-cell assays remain valuable discovery tools but are not yet sufficiently standardized for routine treatment selection [59,60,61,66,67,68,69,70]. Thus, clinically useful biomarker strategies require not only predictive validity but also adequate specimens, reproducible assays, clinically feasible turnaround, interpretable thresholds, and prospective validation in treatment-specific contexts.

Taken together, cervical, endometrial, and ovarian cancers illustrate three distinct patterns of checkpoint sensitivity. Cervical cancer combines persistent HPV-derived antigenicity with frequent immune activation and checkpoint expression, but viral antigens alone are insufficient to overcome defects in antigen presentation, effector-cell access, and adaptive immune suppression; clinical benefit therefore remains strongly dependent on disease setting and treatment backbone. In dMMR/MSI-H endometrial cancer, high neoantigen burden more often coexists with an immune-inflamed microenvironment and a robust predictive enrichment biomarker, providing the most consistent setting for checkpoint sensitivity in gynecologic oncology. Ovarian cancer presents the contrasting pattern: tumor-infiltrating lymphocytes are prognostic, yet effector cells are frequently spatially excluded from malignant epithelium within stromal-, myeloid-, vascular-, and ascites-associated suppressive states, while no broadly reliable biomarker has emerged for unselected checkpoint blockade. Accordingly, immunotherapy efficacy across gynecologic cancers appears to depend not on antigenicity or checkpoint expression alone, but on the convergence of recognizable tumor antigens, intact antigen presentation, effective immune-cell access, reversibility of dominant suppressive programs, clinically informative biomarker selection, and an appropriate therapeutic backbone [4,5,6,7,8,9,10,11,12,13,14,15,16,17,18,19,20,21,32,33,34,35,36,37,38,39,40,41,42,43,44,45,46,52,53,56,57,58,59,60,61,62,63,64,65,66,67,68,69,70,83,84,85,86,87,88,89,90,99,111,112,124,125,126]. Clinically relevant and treatment-informing biomarkers are summarized in Table 2, whereas emerging microenvironmental, resistance, and dynamic monitoring domains are detailed in Supplementary Table S2.

## 3. Cervical Cancer: From Recurrent Disease to Curative-Intent Immunotherapy

### 3.1. Clinical Context and Treatment-Stage Migration

The clinical development of immunotherapy in cervical cancer reflects the interaction between HPV-associated antigenicity and treatment context [32,33,34,35,36,37,42,43,44,45,46,108]. Checkpoint sensitivity is therefore determined not simply by the presence of viral antigens or PD-L1 expression, but by whether the therapeutic backbone can support effective immune recognition and effector-cell activity. Clinically, PD-1/PD-L1 blockade has moved from post-platinum monotherapy to first-line combination therapy and curative-intent chemoradiotherapy [4,5,6,7,8,9,10,11,79,80,99,100,101].

Before the immunotherapy era, the addition of bevacizumab to platinum-based chemotherapy improved survival in persistent, recurrent, or metastatic cervical cancer and established anti-angiogenic therapy as an important systemic treatment component [3]. However, outcomes after progression on platinum-containing therapy remained poor, and second-line chemotherapy produced limited and generally short-lived responses. Immunotherapy development therefore progressed from post-platinum monotherapy to first-line combination therapy and, most recently, to curative-intent chemoradiotherapy. Cervical cancer consequently provides one of the clearest examples of treatment-stage migration in gynecologic immuno-oncology.

### 3.2. Previously Treated Recurrent or Metastatic Disease

Early evidence came from single-arm studies. In KEYNOTE-158, pembrolizumab monotherapy produced an objective response rate of 12.2% in previously treated advanced cervical cancer, and all objective responses occurred in PD-L1-positive tumors; even within the CPS ≥ 1 population, the response rate was 14.6% [99]. Thus, KEYNOTE-158 provided proof of principle and demonstrated the possibility of durable benefit in a minority of patients rather than broad sensitivity to PD-1 blockade. The large proportion of PD-L1-positive patients who did not respond also underscores that PD-L1 enrichment is not equivalent to response prediction.

Cemiplimab provided stronger randomized evidence. In EMPOWER-Cervical 1/GOG-3016/ENGOT-cx9, cemiplimab improved overall survival compared with investigator-choice chemotherapy in patients with recurrent cervical cancer after platinum-based therapy [79]. The final analysis confirmed survival benefit and supported cemiplimab as an active second-line option, including across histologic subgroups [80]. Nivolumab also demonstrated activity in recurrent or metastatic cervical, vaginal, and vulvar cancers in CheckMate 358, and later data evaluating nivolumab with or without ipilimumab suggested that dual checkpoint blockade may increase activity in selected patients, although toxicity and comparative value remain important concerns [100,101]. Collectively, these studies established checkpoint blockade as clinically relevant after platinum failure, but also emphasized that single-agent response rates are insufficient for many patients.

### 3.3. First-Line Immunotherapy for Persistent, Recurrent, or Metastatic Disease

Randomized phase III evidence subsequently established the first-line role of pembrolizumab-containing chemoimmunotherapy in appropriately selected persistent, recurrent, or metastatic cervical cancer. KEYNOTE-826 evaluated pembrolizumab plus platinum-based chemotherapy, with or without bevacizumab, in patients with persistent, recurrent, or metastatic cervical cancer [4]. The trial demonstrated significant improvement in progression-free survival and overall survival, particularly in patients with PD-L1 CPS of at least 1. Final overall survival analysis confirmed sustained benefit, and subgroup analyses showed that benefit was generally preserved across clinically relevant groups, although the magnitude varied by PD-L1 expression, disease setting, histology, prior chemoradiotherapy, geographic region, and bevacizumab use [5,6,7].

KEYNOTE-826 also illustrates how the clinical value of checkpoint blockade depends on treatment context. Its results should not be directly compared with those of later-line checkpoint inhibitor monotherapy studies because the populations, treatment lines, therapeutic backbones, endpoints, and prognosis differ substantially across trials. Accordingly, the apparently greater efficacy observed with first-line combination therapy cannot by itself establish treatment synergy. Potential contributors include chemotherapy-associated antigen release and modulation of suppressive immune populations, as well as bevacizumab-mediated effects on vascular–immune interactions. However, KEYNOTE-826 was not designed to isolate the independent or interactive contributions of chemotherapy, bevacizumab, and pembrolizumab [4,5,6,7,8,99,122]. Bevacizumab should therefore be individualized, particularly in patients with prior pelvic radiotherapy, bleeding or fistula risk, uncontrolled hypertension, thromboembolism, or adjacent-organ invasion.

The BEATcc trial further supported combined chemoimmunotherapy and anti-angiogenic therapy. Atezolizumab added to platinum-based chemotherapy and bevacizumab improved progression-free survival and overall survival compared with chemotherapy plus bevacizumab in untreated metastatic, persistent, or recurrent cervical cancer [8]. These findings are consistent with the biologic rationale that VEGF inhibition may improve vascular–immune crosstalk and immune-cell access, thereby creating a more permissive context for checkpoint blockade [8,122]. BEATcc therefore demonstrates the efficacy of the complete atezolizumab–bevacizumab–chemotherapy strategy rather than proving mechanistic synergy between VEGF and PD-L1 blockade in isolation. Given differences in trial design, biomarker requirements, regulatory status, and local availability, pembrolizumab- and atezolizumab-based regimens should not be considered clinically equivalent in the absence of direct comparative evidence. Pembrolizumab plus chemotherapy, with or without bevacizumab, remains a major evidence-based first-line option for PD-L1-positive disease.

### 3.4. Immunotherapy with Definitive Chemoradiotherapy

The incorporation of immunotherapy into locally advanced cervical cancer represents a major conceptual shift because checkpoint blockade is being combined with definitive, curative-intent chemoradiotherapy rather than used only for recurrent or metastatic disease. Radiotherapy can increase antigen release and immune recognition, but clinical benefit depends on regimen, timing, patient risk profile, and duration of immunotherapy; the discordant results of CALLA and KEYNOTE-A18 demonstrate that immunoradiotherapy should not be treated as a single interchangeable strategy [9,10,11].

The CALLA trial tested durvalumab administered concurrently with and after chemoradiotherapy in previously untreated locally advanced cervical cancer, but did not significantly improve progression-free survival compared with chemoradiotherapy alone [10]. Although negative, CALLA was informative because it showed that immunoradiotherapy benefit cannot be assumed simply because radiation is immunogenic. The discordant results of CALLA and KEYNOTE-A18 cannot be attributed to a single factor. Differences in the enrolled populations, checkpoint agents, treatment schedules, maintenance exposure, and other trial-design features preclude a direct mechanistic explanation and argue against assuming a uniform class effect.

KEYNOTE-A18 established pembrolizumab plus definitive chemoradiotherapy followed by pembrolizumab maintenance as an evidence-based strategy for the high-risk locally advanced cervical cancer population represented in the trial [9,11]. The initial analysis demonstrated improved progression-free survival, and subsequent overall survival analysis confirmed a clinically meaningful survival advantage [9,11]. KEYNOTE-A18 therefore extends checkpoint blockade from metastatic disease into curative-intent treatment. The clinical implications extend beyond efficacy: patients who may be cured also require careful attention to late immune-related toxicity, survivorship, fertility counseling, ovarian and sexual function, and the attribution of symptoms that may overlap with pelvic radiation effects.

Patient selection should remain aligned with the eligibility and risk characteristics of KEYNOTE-A18, because the benefit of adding pembrolizumab has not been established across all locally advanced cervical cancer populations or with other immunoradiotherapy regimens. The negative CALLA trial and positive KEYNOTE-A18 results together argue against indiscriminate class-wide assumptions [9,10,11]. In practice, the benefits of adding pembrolizumab should be weighed against baseline autoimmune disease, pulmonary or hepatic comorbidity, feasibility of prolonged maintenance therapy, and the ability to evaluate immune-related symptoms that resemble radiation toxicity. Future studies should determine whether all high-risk patients require the full planned maintenance duration or whether early molecular, virologic, or radiographic response can support de-escalation.

### 3.5. HPV-Directed and Post-ICI Strategies

Beyond conventional checkpoint blockade, HPV-directed vaccines and cellular therapies provide proof of concept for antigen-specific immune targeting in cervical cancer. Therapeutic HPV vaccines, TIL therapy, and HPV E7-directed TCR strategies have shown activity in selected advanced HPV-associated cancers, but their development is constrained by early-phase evidence, specialized manufacturing, lymphodepletion requirements, and treatment-related toxicity [102,103,104].

Dual checkpoint targeting is another emerging strategy. Cadonilimab, a PD-1/CTLA-4 bispecific antibody, has progressed from early-phase activity to phase III evidence: COMPASSION-16 showed improved progression-free and overall survival when cadonilimab was added to platinum-based chemotherapy, with or without bevacizumab, in persistent, recurrent, or metastatic cervical cancer [81,127]. Its comparative value relative to established pembrolizumab- or atezolizumab-based regimens, biomarker selection, global applicability, and activity after prior PD-1 exposure remain unresolved.

As pembrolizumab moves into first-line systemic and curative-intent treatment, post-ICI sequencing has become the dominant unresolved clinical question. Whether subsequent therapy should rely on renewed immune targeting or on treatments with ICI-independent activity remains uncertain [12,101,102,103,104,128]. Future trials should therefore specifically enroll prior-ICI populations and test sequencing strategies rather than extrapolate from ICI-naive cohorts.

## 4. Endometrial Cancer: A Biomarker-Driven Model

### 4.1. Molecular Classification and Clinical Implications

Endometrial cancer provides the clearest example of biomarker-enriched checkpoint sensitivity in gynecologic oncology. In dMMR/MSI-H disease, high mutational antigenicity is coupled with a comparatively immune-inflamed phenotype and a clinically validated enrichment biomarker, helping explain the large and reproducible treatment effects observed across recurrent and first-line settings [15,16,17,18,19,39,40,47,48,49,50,51,52,53,109]. By contrast, pMMR/MSS disease is biologically heterogeneous and generally requires combination strategies or additional selection markers. POLE-mutated tumors are highly immunogenic but usually have an excellent prognosis, making treatment de-escalation rather than routine immunotherapy intensification the more relevant clinical question [41,50,51,55].

### 4.2. PD-1 Blockade in Recurrent dMMR/MSI-H Disease

The first major success of immunotherapy in endometrial cancer occurred in recurrent dMMR/MSI-H disease. In KEYNOTE-158, pembrolizumab produced an ORR of approximately 48% in previously treated MSI-H/dMMR advanced endometrial cancer, with durable responses among responders [52,53]. Dostarlimab provided complementary evidence in GARNET, with an ORR of approximately 43.5% in dMMR/MSI-H advanced or recurrent endometrial cancer after platinum-based therapy [109]. These studies establish dMMR/MSI-H as a clinically useful predictive enrichment biomarker for PD-1 blockade, but the response rates also demonstrate that biomarker positivity does not guarantee an objective response.

Several clinical questions remain. Primary resistance occurs despite dMMR/MSI-H status and may reflect impaired antigen processing or HLA presentation, altered interferon signaling, immune exclusion, suppressive myeloid or stromal programs, and intratumoral heterogeneity [35,52,53,94,95,110]. Importantly, the relative contribution of these mechanisms in dMMR/MSI-H endometrial cancer remains incompletely defined, and no resistance biomarker currently supersedes MMR status for routine treatment selection. Optimal treatment duration is not firmly established, and the role of rechallenge after prior PD-1 exposure is unclear. These issues are becoming more important as PD-1/PD-L1 inhibitors move into first-line therapy, because later-line decisions will increasingly involve patients previously exposed to immunotherapy [15,16,17,18,19,20,21].

### 4.3. Pembrolizumab Plus Lenvatinib in pMMR/MSS Recurrent Disease

For pMMR/MSS recurrent endometrial cancer, pembrolizumab plus lenvatinib is a phase III-supported post-platinum treatment option. Lenvatinib targets angiogenic and growth factor pathways and may enhance antitumor immunity by counteracting VEGF-mediated suppression and modifying the tumor microenvironment. In KEYNOTE-775, lenvatinib plus pembrolizumab improved progression-free survival and overall survival compared with physician-choice chemotherapy in previously treated advanced endometrial cancer, including the pMMR population [13,54]. This provided an important treatment option for patients who historically had limited benefit from conventional chemotherapy after platinum failure.

Toxicity is central to the clinical use of this regimen. Hypertension, gastrointestinal symptoms, fatigue, thyroid dysfunction, renal dysfunction, dermatologic toxicity, and immune-mediated events require proactive monitoring and early intervention. Lenvatinib interruption and dose reduction are common and form an integral part of toxicity management; they do not necessarily indicate loss of treatment efficacy or require permanent discontinuation. The negative first-line ENGOT-en9/LEAP-001 trial, in which lenvatinib plus pembrolizumab did not replace carboplatin-paclitaxel as a universal first-line option, further emphasizes that activity in the post-platinum setting does not automatically translate into superiority over chemotherapy in untreated disease [14].

Pembrolizumab–lenvatinib should therefore be regarded as an evidence-supported post-platinum option rather than as a universally optimal strategy. Its substantial toxicity must be weighed against molecularly or clinically selected alternatives, and no direct head-to-head evidence demonstrates superiority over all such options [13,20,21,54].

### 4.4. First-Line Chemoimmunotherapy and Maintenance Therapy

The major recent shift in endometrial cancer is the movement of PD-1/PD-L1 blockade into first-line treatment for primary advanced or recurrent disease. Multiple randomized phase III trials—including NRG-GY018, RUBY, DUO-E, and AtTEnd—have demonstrated improved progression-free survival with chemoimmunotherapy-based strategies, with the largest and most consistent treatment effects observed in dMMR/MSI-H tumors [15,16,17,18,19]. RUBY also demonstrated an overall survival benefit in the overall population [16,17]. Together, these trials provide practice-informing phase III evidence for induction chemoimmunotherapy followed by protocol-defined maintenance as an effective first-line framework, while highlighting greater biological heterogeneity and a smaller average magnitude of benefit in pMMR/MSS disease.

An important limitation is that induction and maintenance were generally embedded within the same experimental strategy rather than being independently randomized. Their relative contributions therefore remain uncertain, particularly in multi-component regimens such as DUO-E. Future studies should determine which patients require chemotherapy, prolonged maintenance, or additional targeted therapy rather than assuming that the complete trial regimen is necessary for every patient.

### 4.5. Decision-Making in an Era of Multiple Options

For dMMR/MSI-H disease, the remaining question is increasingly whether all patients require the complete chemoimmunotherapy strategy. Evidence for PD-1 monotherapy derives mainly from previously treated advanced or recurrent populations [15,16,17,18,19,52,53,109]. Chemotherapy-free PD-1 blockade may be attractive for selected patients because it could reduce chemotherapy-related treatment burden, but omission of chemotherapy in treatment-naïve advanced disease remains unproven. No prospective trial has yet established a chemotherapy-free immunotherapy strategy as non-inferior to standard first-line therapy with superior tolerability; this question requires dedicated de-escalation or non-inferiority trials incorporating toxicity and patient-reported outcomes.

For pMMR/MSS disease, benefit from first-line chemoimmunotherapy is more heterogeneous, and selection should extend beyond MMR status to molecular subtype, HER2 status, hormone-receptor expression, DNA-damage-response features, disease tempo, prior therapy, comorbidity, and patient preference [20,21]. Importantly, the evidence supporting pembrolizumab–lenvatinib was generated largely before routine first-line ICI exposure, and its efficacy after prior PD-1/PD-L1-containing chemoimmunotherapy remains insufficiently defined. Consequently, its current post-platinum role should not be extrapolated automatically to the increasingly common post-ICI population [13,15,16,17,18,19,54]. Regulatory indications and access vary by region and agent.

### 4.6. Earlier-Stage Disease and De-Escalation

As immunotherapy becomes standard in advanced and recurrent disease, its role in earlier-stage and adjuvant settings is under active investigation. KEYNOTE-B21 did not improve disease-free survival in the all-comer population. A favorable DFS signal was observed in the preplanned dMMR subgroup [82]. However, even prespecified subgroup findings should be interpreted in the context of the negative primary all-comer analysis, subgroup sample size and precision, and the trial’s prespecified statistical testing strategy. Accordingly, the dMMR finding should be regarded as supportive and hypothesis-generating rather than independently practice-defining. These results argue against all-comer adjuvant immunotherapy and support prospective confirmation in molecularly selected populations.

The RAINBO program reflects this direction by testing adjuvant strategies according to molecular class [129]. The excellent prognosis associated with pathogenic POLE exonuclease-domain mutations makes this subgroup a priority for adjuvant de-escalation research. However, treatment omission or reduction should follow validated molecular risk algorithms or prospective protocols rather than immunogenicity or POLE status in isolation. dMMR tumors raise the question of whether adjuvant immunotherapy improves disease control in truly high-risk patients. p53-abnormal tumors may require systemic intensification or targeted approaches in selected settings, whereas NSMP tumors require further risk stratification beyond histology alone. This molecularly stratified approach should become central to future endometrial cancer trial design.

The optimal duration of maintenance immunotherapy also remains undefined, and prospective de-escalation studies should determine whether response depth or validated molecular residual-disease measures can support individualized treatment discontinuation [15,16,17,18,19,120,121].

## 5. Ovarian Cancer: From Broadly Negative Trials to a Biomarker-Selected Exception

### 5.1. The Disconnect Between Prognostic Immunity and Therapeutic Benefit

Ovarian cancer provides the clearest example of the disconnect between prognostic immune infiltration and checkpoint sensitivity. Although tumor-infiltrating lymphocytes are associated with favorable outcomes, effector cells may remain spatially excluded from malignant epithelial nests within stromal-, myeloid-, vascular-, and ascites-associated suppressive environments [56,57,58,59,60,61,62,63,64,65,66,67,68,69,70]. Combined with the absence of a broadly reliable predictive biomarker, this biology helps explain why multiple unselected checkpoint inhibitor trials have been negative despite evidence that antitumor immunity is prognostically relevant [83,84,85,86,87,111,112].

### 5.2. Single-Agent Checkpoint Blockade and Chemotherapy Combinations

KEYNOTE-100 evaluated pembrolizumab monotherapy in recurrent ovarian cancer and showed limited overall activity, although a subset of patients achieved responses [111]. Exploratory analyses suggested that immune and molecular features may influence outcome, but no single biomarker has been sufficiently robust for routine selection in unselected ovarian cancer [112]. JAVELIN Ovarian 200 tested avelumab alone or with pegylated liposomal doxorubicin in platinum-resistant or platinum-refractory ovarian cancer and did not improve progression-free or overall survival compared with chemotherapy alone [84]. These results indicated that simply adding PD-L1 blockade to chemotherapy in resistant disease does not reliably overcome ovarian cancer immune resistance.

More recently, ENGOT-ov65/KEYNOTE-B96 provided the first phase III evidence that an immune checkpoint inhibitor-based regimen can improve survival in platinum-resistant recurrent ovarian cancer. In this randomized, double-blind trial, 643 patients who had received one or two prior systemic regimens were assigned to pembrolizumab or placebo plus weekly paclitaxel, with or without bevacizumab [85]. Among patients with PD-L1 CPS ≥ 1 disease, pembrolizumab improved median PFS from 7.2 to 8.3 months (HR 0.72), corresponding to a modest absolute median PFS gain of 1.1 months, while median OS improved from 14.0 to 18.2 months (HR 0.76), an absolute difference of 4.2 months [85]. Thus, the statistically significant PFS result should not be interpreted as a large absolute delay in progression. The regimen therefore represents an evidence-based, biomarker- and backbone-specific treatment option rather than a universally preferred standard, and its clinical use should be individualized according to regulatory availability, toxicity, treatment burden, quality of life, prior treatment, bevacizumab eligibility, and available alternatives.

The first-line setting has been similarly difficult. JAVELIN Ovarian 100 evaluated avelumab with and/or after platinum-based chemotherapy in previously untreated epithelial ovarian cancer and was stopped for futility [83]. This result showed that avelumab-containing chemoimmunotherapy or maintenance did not improve outcomes in an unselected first-line population. It therefore argues against assuming that a platinum–taxane backbone can reliably sensitize unselected ovarian cancer to PD-L1 blockade. Accordingly, checkpoint inhibitors should still not be regarded as routine treatment for unselected ovarian cancer. Their current use should be confined to validated biomarker- or regimen-defined settings, including tumor-agnostic MSI-H/dMMR or TMB-high indications and pembrolizumab plus weekly paclitaxel, with or without bevacizumab, for PD-L1 CPS ≥1 platinum-resistant disease after one or two prior systemic regimens [85,119].

### 5.3. Anti-Angiogenic and PARP Inhibitor Combinations

Anti-angiogenic therapy is a rational partner for immunotherapy because VEGF-driven vascular and immune dysfunction can restrict effector-cell trafficking, impair dendritic-cell function, promote T-cell dysfunction, and support myeloid-mediated suppression; vascular normalization may therefore create a more permissive microenvironment for checkpoint blockade [122]. Despite a strong biological rationale for VEGF–ICI combinations, IMagyn050 showed that adding atezolizumab to first-line carboplatin–paclitaxel plus bevacizumab did not improve PFS in either the intention-to-treat or PD-L1-positive populations, and subsequent analyses did not establish OS or patient-reported outcome benefit [86,87]. The contrast between BEATcc and IMagyn050 illustrates that the same broad mechanistic rationale—vascular normalization combined with checkpoint blockade—does not translate uniformly across tumor lineages, emphasizing the importance of disease-specific immune architecture, biomarker context, and treatment backbone [8,86,87,122].

PARP inhibition also has a strong mechanistic rationale for combination with checkpoint blockade through DNA-damage-induced innate immune signaling, but encouraging early-phase signals have not resolved whether ICI adds clinically meaningful benefit beyond contemporary PARP-based treatment [123,124,125,126].

Phase III trials have produced positive but difficult-to-deconvolute results. DUO-O improved PFS with a durvalumab–olaparib-containing strategy, but the control arm did not include PARP inhibitor maintenance, preventing isolation of the independent contribution of PD-L1 blockade [88]. Similarly, KEYLYNK-001 improved PFS with pembrolizumab–olaparib maintenance, but the absence of an olaparib-alone control prevented attribution of benefit specifically to pembrolizumab [89].

FIRST/ENGOT-OV44 reported a statistically significant but modest PFS improvement with dostarlimab added to chemotherapy and niraparib-based maintenance, without an established OS advantage in the reported analysis [90]. Taken together, these studies support continued evaluation of PARP–ICI strategies but also show why contemporary active controls and component-informative designs are essential before the incremental value of checkpoint blockade can be established.

### 5.4. Mechanisms That Require More than Checkpoint Blockade

Ovarian cancer likely requires strategies that address the dominant mechanism of immune exclusion rather than checkpoint signaling alone. Mechanistic classification may therefore provide a framework for combination development: tumors characterized by abnormal vasculature and impaired lymphocyte trafficking may be better suited to vascular-normalizing strategies; fibroblast- or stroma-dominant tumors may require remodeling of physical or chemokine-mediated barriers to T-cell entry; and myeloid-dominant tumors may require macrophage- or MDSC-directed approaches. By contrast, tumors with major defects in antigen processing or presentation may remain resistant even when lymphocytes are present and are unlikely to be rescued simply by intensifying PD-1/PD-L1 blockade [32,33,34,35,57,59,60,61,62,67,68,69,70,94,95,110,122]. These phenotypes are not yet validated for routine treatment selection, but they provide a rationale for biomarker-enriched, mechanism-matched trials rather than empirical all-comer combinations.

Spatial and single-cell studies are reshaping this field by showing that ovarian tumors cannot be classified simply as immune-rich or immune-poor. The location, phenotype, and functional state of immune cells may be as important as their abundance [59,60,61,66,67,68,69,70]. These approaches may eventually support composite biomarkers that distinguish immune-inflamed, immune-excluded, myeloid-dominant, ascites-driven, and antigen-presentation-defective tumors and thereby enrich trials for interventions matched to the dominant resistance mechanism. However, such classifications require prospective analytical and clinical validation before they can guide routine treatment selection.

### 5.5. Future Directions in Ovarian Cancer

Future ovarian cancer immunotherapy should focus on whether prospectively defined resistance states can identify patients who benefit from mechanism-matched immune combinations. Rather than treating immune exclusion as a single phenotype, trials should distinguish dominant vascular, stromal, myeloid, ascites-associated, and antigen-presentation defects and test interventions matched to those mechanisms [35,62,63,64,65,66,67,68,69,70,110].

KEYNOTE-B96 supports pembrolizumab as a biomarker- and backbone-specific treatment option in PD-L1 CPS ≥ 1 platinum-resistant disease, but the remaining clinical priorities are narrower: refining selection beyond CPS and defining treatment after prior PARP inhibitor, bevacizumab, or ICI exposure, including sequencing relative to FRα-directed ADCs [22,23,24,25,26,27,85]. Outside this setting, large unselected trials are unlikely to resolve ovarian cancer immune resistance; future studies should therefore use contemporary active controls and prospectively enriched populations.

## 6. Gestational Trophoblastic Neoplasia and Rare Gynecologic Malignancies

### 6.1. Gestational Trophoblastic Neoplasia

GTN combines potential immunogenicity from paternal alloantigens with trophoblastic immune-tolerance programs, including PD-L1 and HLA-G expression [71,72,73,74]. This provides a strong rationale for checkpoint blockade, but its clinical role must be interpreted against the exceptionally high cure rates achieved with risk-adapted chemotherapy [75,76,77,78,130].

Because risk-adapted chemotherapy cures most GTN, checkpoint blockade should not be viewed as a replacement for established treatment and is currently most relevant to multidrug-resistant, relapsed, or selected metastatic disease [75,76,77,78,130].

Clinical evidence began with reports of pembrolizumab producing durable complete responses in multidrug-resistant GTN [131]. The phase II CAP 01 study demonstrated clinically relevant activity of camrelizumab plus apatinib in high-risk chemorefractory or relapsed GTN [93]. A multicenter retrospective study suggested that anti-PD-1 therapy plus chemotherapy was associated with higher complete response and overall response rates than anti-PD-1 monotherapy in high-risk chemorefractory or relapsed GTN, although combination therapy produced more grade 3–4 treatment-related adverse events [132]. These data support further prospective testing of combination strategies in heavily pretreated or high-risk settings.

Prospective evidence is provided by TROPHIMMUN, in which avelumab achieved hCG normalization in approximately half of patients resistant to single-agent chemotherapy but showed limited activity after polychemotherapy failure, suggesting that treatment line and disease burden may influence checkpoint sensitivity [91,92]. TROPHAMET subsequently reported a 96.2% hCG-normalization rate with first-line avelumab plus methotrexate in low-risk disease, but its small nonrandomized design and the high curability of low-risk GTN make routine early-line immunotherapy difficult to justify without comparative evidence [133].

The principal unresolved questions in GTN are therefore the optimal timing and duration of checkpoint blockade and its integration with established chemotherapy and surgery. hCG may permit response-adapted treatment in most GTN, whereas PSTT and ETT require separate evaluation because biochemical response may not accurately reflect disease burden [75,76,77,78,91,92,93,130,131,132,134]. Prospective studies should prioritize these disease-specific treatment questions rather than routine expansion of ICI into highly curable standard-risk populations.

### 6.2. Vulvar, Vaginal, and Clear Cell Gynecologic Cancers

Evidence for checkpoint blockade in vulvar and vaginal cancers remains limited. HPV-associated tumors provide a biological rationale for cautious extrapolation from cervical cancer, and pembrolizumab or nivolumab has shown durable activity in a minority of previously treated patients [100,105,106]. However, prospective disease-specific evidence is sparse, and PD-L1 expression alone is insufficient for treatment selection [118].

Gynecologic clear-cell cancers represent a signal-generating histology-specific context. PEACOCC and MoST-CIRCUIT reported encouraging checkpoint activity in predominantly pMMR ovarian and endometrial clear-cell cancers [113,114]. However, both datasets are nonrandomized and do not establish clear-cell morphology itself as a validated predictive biomarker; prospective histology-specific confirmation remains necessary.

### 6.3. Uterine Sarcomas and Other Rare Non-Epithelial Tumors

Immunotherapy in uterine sarcomas remains histology- and biomarker-dependent. In SARC028, pembrolizumab activity was concentrated in selected sarcoma histologies and no objective responses were observed in the leiomyosarcoma cohort, cautioning against broad extrapolation to uterine sarcomas [115,116]. In practice, checkpoint blockade is most relevant to tumor-agnostic biomarker-defined situations such as MSI-H/dMMR or TMB-high disease rather than routine histology-based treatment; isolated case reports do not establish broader efficacy [117].

More broadly, the principal priority for rare gynecologic malignancies is rarity-aware prospective evidence generation in biologically coherent cohorts rather than cross-lineage extrapolation. Harmonized pathology and molecular characterization should support disease-appropriate endpoints, with GTN, clear-cell tumors, and sarcomas treated as distinct development models rather than as a single “rare tumor” category [75,76,77,78,91,92,93,105,106,113,114,115,116,117,130,131,132,134]. Shared rarity should not be confused with shared immune biology.

## 7. Combination Strategies, Toxicity, and Future Directions

**Figure 2:**
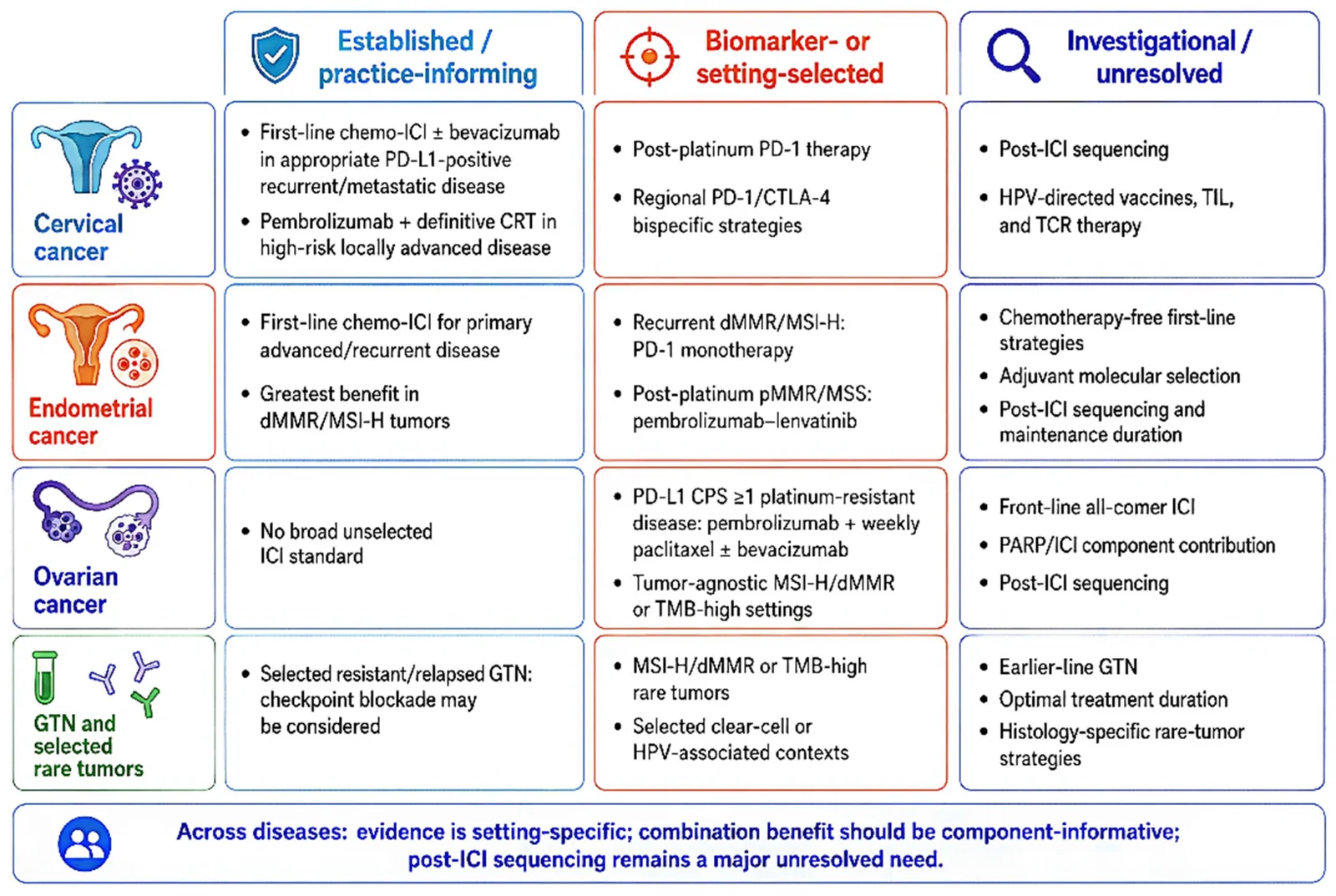
Evidence-based clinical positioning of immunotherapy across gynecologic cancers. Clinical applications are categorized as established/practice-informing, biomarker- or setting-selected, or investigational/unresolved according to the maturity and context of the supporting evidence. These categories reflect a pragmatic clinical interpretation rather than a formal guideline grading system. The framework emphasizes major disease-specific treatment roles and unresolved clinical questions rather than individual trial-level efficacy estimates. Abbreviations: CPS, combined positive score; CTLA-4, cytotoxic T-lymphocyte-associated protein 4; dMMR, mismatch repair-deficient; GTN, gestational trophoblastic neoplasia; HPV, human papillomavirus; ICI, immune checkpoint inhibitor; MSI-H, microsatellite instability-high; MSS, microsatellite stable; PARP, poly(ADP-ribose) polymerase; PD-1, programmed cell death protein 1; PD-L1, programmed death-ligand 1; pMMR, mismatch repair-proficient; TCR, T-cell receptor; TIL, tumor-infiltrating lymphocyte; TMB, tumor mutational burden.

A simplified evidence-based framework for the clinical positioning of immunotherapy across gynecologic cancers is shown in Figure 2.

### 7.1. Principles of Rational Combination Therapy

Combination therapy should be judged by whether each component addresses a defined resistance mechanism and adds clinically meaningful benefit over a contemporary control [28,29,30,31,32,33,34,35,122,123]. Interpretation should remain specific to tumor lineage, biomarker-defined population, treatment line, and therapeutic backbone rather than assuming a class-wide effect.

Rational development therefore requires mechanism-based patient enrichment and trial designs capable of isolating the incremental contribution of immunotherapy. When an experimental strategy adds multiple active agents to a control that does not contain the contemporary non-ICI standard, a positive result establishes the value of the overall strategy but not necessarily the independent contribution of checkpoint blockade. Future combinations should therefore be mechanism-matched rather than empirically additive and, where feasible, evaluated with contemporary active controls or component-informative designs. These principles and their disease-specific applications are summarized in Table 3. Accordingly, positive strategy-level evidence should be distinguished from evidence that establishes the independent clinical contribution of checkpoint blockade.

### 7.2. Toxicity, Reproductive Health, and Survivorship

As immunotherapy moves from salvage therapy to first-line, maintenance, and curative-intent settings, toxicity management becomes increasingly important. Immune-related adverse events can involve almost any organ system and may occur during treatment or after discontinuation [137,138,139,140,141,143,144,145]. Safe delivery requires early recognition, severity grading, treatment interruption when indicated and corticosteroids for clinically significant events [137,138,139,140,141].

Clinical monitoring should be tailored to the treatment backbone. In cervical cancer, immune-mediated bowel, bladder, hepatic, and constitutional symptoms may overlap with chemoradiotherapy toxicity, complicating attribution [9,10,11,137,138,139,140,141,143,144,145]. In endometrial cancer, pembrolizumab plus lenvatinib combines immune-related adverse events with hypertension, gastrointestinal toxicity, thyroid dysfunction, renal effects, and fatigue [13,14,137,138,139,140,141]. In ovarian cancer, multi-agent induction and maintenance regimens may compound cytopenias, fatigue, gastrointestinal toxicity, vascular events, and renal dysfunction [22,23,24,25,26,27,83,84,85,86,87,88,89,90,124,125,126,137,138,139,140,141].

In palliative settings, toxicity should be interpreted cumulatively rather than drug by drug. Multi-agent regimens may combine immune-mediated adverse events with chemotherapy-related cytopenias, neuropathy and fatigue, VEGF-pathway toxicity such as hypertension, bleeding or thrombosis, and PARP- or TKI-related hematologic, gastrointestinal and constitutional toxicity. Even when each component is individually manageable, dose interruptions, repeated monitoring, corticosteroid treatment, additional clinic visits, and persistent low-grade symptoms can materially reduce quality of life. Accordingly, modest PFS gains should be weighed against overall survival, symptom control, patient-reported outcomes, treatment burden, and patient preference, particularly when the therapeutic intent is palliative.

Financial toxicity should also be considered part of the cumulative treatment burden of immunotherapy. Beyond drug-acquisition costs, prolonged or multi-agent regimens may require repeated biomarker testing, infusions, laboratory and imaging surveillance, management of immune-related or vascular adverse events, travel, and time away from work. These burdens may be amplified in resource-constrained settings, where access to checkpoint inhibitors, companion diagnostics, infusion infrastructure, and specialist toxicity management may be limited. Accordingly, treatment selection—particularly in palliative settings—should consider affordability, feasibility, treatment duration, and local access alongside efficacy, physical toxicity, quality of life, and patient preference. Prospective trials and real-world studies should, where feasible, incorporate resource utilization and patient-reported financial burden into assessment of treatment value.

Reproductive and survivorship considerations are particularly important as immunotherapy enters first-line and curative-intent settings. Because PD-1/PD-L1 signaling contributes to maternal–fetal tolerance, checkpoint inhibitors are generally avoided during pregnancy [142]. Reproductive-age patients should receive counseling regarding contraception, fertility preservation, endocrine immune-related adverse events, uncertainty surrounding ovarian reserve, and the timing of pregnancy after treatment. Trials should prospectively evaluate late toxicity, sexual health, ovarian and endocrine function, quality of life, and patient-reported outcomes rather than treating these measures as secondary endpoints.

### 7.3. Resistance, Response Assessment, and Sequencing

Sequencing decisions should be guided not only by tumor type but also by the timing and probable mechanism of resistance. Primary resistance may reflect absent immune recognition, antigen-presentation failure, or an excluded and suppressive microenvironment. Acquired resistance after an initial period of clinical benefit may arise through immunoediting and clonal selection of less immunogenic tumor cells, loss of antigen processing or HLA class I presentation, alterations affecting B2M or interferon/JAK signaling, adaptive activation of alternative inhibitory pathways, or remodeling toward a more stromal- or myeloid-suppressive microenvironment [94,95,110]. These mechanisms may provide a biological rationale for different post-ICI strategies, but most have been defined in broader solid-tumor studies and have not yet been prospectively validated for treatment selection in gynecologic cancers. Mechanistically informed post-ICI studies should therefore incorporate paired pretreatment and progression biopsies when feasible, together with longitudinal ctDNA or other blood-based profiling, to determine whether resistance reflects tumor-intrinsic evolution, microenvironmental adaptation, or both. Such studies may help identify patients more likely to benefit from immune rechallenge, alternative checkpoint targeting, microenvironmental remodeling, or a switch to non-immune therapies.

Immune checkpoint inhibitors may produce delayed responses, mixed responses, and rare pseudoprogression. iRECIST standardizes these patterns in clinical trials, but treatment beyond radiographic progression in routine practice should be restricted to clinically stable patients without rapid or organ-threatening deterioration [136]. GTN is distinct because serum hCG often provides early biochemical response assessment, although imaging and clinical judgment remain essential in PSTT and ETT, in which hCG may underestimate disease burden.

Until prospective post-ICI data are available, a pragmatic approach is to avoid routine PD-1/PD-L1 rechallenge outside a clinical trial and to prioritize therapies with independent activity that do not depend on renewed checkpoint sensitivity. In cervical cancer, options may include tisotumab vedotin where available, chemotherapy with or without bevacizumab if not previously exhausted and clinically appropriate, local treatment for selected oligoprogression, and clinical trials of HPV-directed or cellular therapies [4,5,6,7,8,9,10,11,12,101,102,103,104,128]. In endometrial cancer, treatment after frontline ICI should be guided by MMR and molecular subtype, HER2 and hormone-receptor status, prior immune response, residual toxicity, and disease tempo; the activity of pembrolizumab-lenvatinib after prior PD-1/PD-L1 exposure remains insufficiently defined [13,14,15,16,17,18,19,20,21,53,54,82,109,129]. In ovarian cancer, post-ICI decisions should prioritize established biomarker-directed and cytotoxic options according to FRα, BRCA/HRD status, and prior PARP inhibitor or bevacizumab exposure [22,23,24,25,26,27,83,84,85,86,87,88,89,90,124,125,126]. These recommendations represent a pragmatic sequencing framework rather than prospectively validated standards.

### 7.4. Evidence Interpretation, Dynamic Monitoring, and Future Trial Design

Dynamic biomarkers may support early response assessment and decisions regarding treatment duration. Circulating tumor DNA (ctDNA) can detect molecular residual disease and relapse risk in uterine and ovarian cancers, whereas hCG already functions as a disease-specific dynamic marker in most GTN [120,121,130]. Potential applications include identifying early nonresponse, clarifying equivocal imaging findings, and guiding treatment escalation, de-escalation, or discontinuation. However, prospective validation is required before ctDNA-guided immunotherapy decisions can be adopted routinely.

Four unresolved clinical questions should now take priority across gynecologic cancers. First, patient selection should determine who truly requires immunotherapy, who may safely receive treatment de-escalation, and who is unlikely to benefit despite apparently favorable biomarkers. Second, prospective evidence is needed to define treatment after prior ICI exposure, including the roles of rechallenge, ADCs, targeted therapy, cellular approaches, and non-immune strategies. Third, multi-component regimens should establish the independent contribution and optimal duration of checkpoint blockade using contemporary active controls and, where feasible, component-informative designs. Fourth, prospectively defined resistance mechanisms should be linked to treatment selection, so that immune-excluded, stromal-, myeloid-, vascular-, or antigen-presentation-defective tumors can be tested in mechanism-matched rather than empiric all-comer trials. Longitudinal translational sampling and disease-specific stratification should support these priorities rather than constitute endpoints in themselves [4,5,6,7,8,9,10,11,12,13,14,15,16,17,18,19,20,21,22,23,24,25,26,27,35,62,63,64,65,66,67,68,69,70,81,91,92,93,94,95,96,97,98,102,103,104,105,106,107,108,110,113,114,115,116,117,118,119,120,121,122,123,127,128,130,131,132,133,134,135,136].

Interpretation of immunotherapy evidence should distinguish clinical maturity from statistical positivity and biological plausibility. Subgroup and multiple-endpoint findings require particular caution. Their evidentiary weight depends on prespecification, statistical precision, testing hierarchy, alpha allocation, and multiplicity control. Nominally significant findings outside a protected statistical framework should generally be regarded as supportive or hypothesis-generating rather than independently confirmatory. This distinction is particularly important when the primary analysis is negative; for example, the favorable preplanned dMMR signal in KEYNOTE-B21 does not independently establish adjuvant immunotherapy as a standard in this subgroup [82].

Cross-trial comparisons in this review are therefore qualitative rather than indirect treatment comparisons. Differences in disease setting, prior therapy, biomarker selection, treatment backbone, comparator, endpoint definition, follow-up, and post-progression therapy can materially affect observed efficacy estimates and preclude reliable ranking of regimens or inference of pharmacologic synergy. Across randomized phase III studies, the most consistent benefits have been observed in established cervical cancer settings and biomarker-enriched endometrial cancer, particularly dMMR/MSI-H disease, whereas immunotherapy trials in unselected ovarian cancer have been predominantly negative or have shown modest effects. KEYNOTE-B96 represents a biomarker- and regimen-defined exception, but the absolute median PFS gain in PD-L1 CPS ≥ 1 disease was only 1.1 months, despite a favorable HR of 0.72 [83,84,85,86,87,88,89,90,124,125,126]. Because of substantial heterogeneity in tumor lineage, treatment line, biomarker definition, control backbone, maintenance strategy, and endpoint structure, we did not calculate a pooled summary effect. Regulatory authorization likewise should not be considered synonymous with a universally preferred standard of care; clinical positioning also depends on evidence maturity, comparator relevance, guideline recommendations, biomarker-defined eligibility, toxicity, available alternatives, access, and patient characteristics.

Hazard ratios should also be interpreted in a clinical context rather than as constant relative risk reductions throughout follow-up. A conventional constant-effect interpretation assumes proportional hazards, whereas immunotherapy effects may vary over time and survival curves may show delayed or changing separation. Because this review relies on published aggregate data, the proportional-hazards assumption cannot be independently assessed for individual trials. Relative effects should therefore be interpreted alongside absolute measures such as differences in median PFS or OS, landmark survival rates, response durability, patient-reported outcomes, toxicity, and treatment burden. More broadly, statistical significance should not be equated automatically with clinical meaningfulness, which depends on the magnitude and durability of absolute benefit, effects on survival and quality of life, and the effectiveness and tolerability of available alternatives.

De-escalation is a related implementation question in biomarker-defined populations with high checkpoint sensitivity. Less-intensive strategies should be evaluated in prospectively designed non-inferiority trials with predefined margins and comparative toxicity and patient-reported outcomes; similar efficacy estimates observed post hoc in superiority trials should not be interpreted as evidence of non-inferiority.

Formal cost-effectiveness is related to, but distinct from, patient-level financial toxicity. Economic evaluations of immunotherapy-based regimens in cervical and endometrial cancer demonstrate that value may vary according to drug prices, biomarker-defined treatment effects, treatment duration, downstream care, health-state utilities, and healthcare-system-specific willingness-to-pay thresholds [146,147]. Accordingly, a regimen that is economically favorable in one healthcare system may not provide equivalent value in another, and neither regulatory approval nor clinical efficacy should be assumed to imply cost-effectiveness. This consideration is particularly relevant to multi-agent regimens and prolonged maintenance strategies. Future trials and implementation studies should therefore incorporate resource utilization and, where feasible, quality-adjusted outcomes alongside efficacy, toxicity, and patient-reported outcomes.

Finally, conventional PFS and OS endpoints should be complemented by duration of response, treatment-free interval, late toxicity, patient-reported outcomes, fertility and sexual health, endocrine function, and molecular response [91,92,93,120,121,130,131,132,134,136,137,138,139,140,141,142,143,144,145]. Generalizability should also be considered when translating pivotal trial results into routine practice. Many immunotherapy trials preferentially enroll patients with good performance status, adequate hematologic, hepatic, and renal function, controlled comorbidities, and protocol-defined limits on prior therapy or prior ICI exposure. Patients with poor performance status, clinically significant organ dysfunction, active autoimmune disease, bowel compromise, complex prior pelvic radiotherapy, or heavy pretreatment remain underrepresented. Prospective real-world cohorts should therefore complement rather than replace randomized evidence by defining effectiveness, tolerability, treatment modification and discontinuation, financial toxicity and access, and post-progression sequencing in populations insufficiently represented in pivotal trials.

## 8. Conclusions

Immunotherapy has become an integral but highly context-dependent component of gynecologic oncology. Across tumor types, checkpoint sensitivity is determined not by antigenicity or PD-L1 expression alone, but by the interaction of tumor lineage, antigen presentation, immune-cell access, suppressive microenvironmental programs, biomarker-defined selection, and treatment backbone. This framework helps explain why biomarker-enriched endometrial cancer and selected cervical cancer settings show reproducible benefit, whereas most unselected ovarian cancer strategies have been unsuccessful despite prognostically relevant immune infiltration. The KEYNOTE-B96 findings define a biomarker and backbone-specific exception in platinum-resistant ovarian cancer rather than overturning this broader pattern. GTN and selected rare gynecologic malignancies further demonstrate that lineage-specific immune biology can identify therapeutic opportunities, although the maturity of supporting evidence varies substantially across these settings [4,5,6,7,8,9,10,11,12,13,14,15,16,17,18,19,20,21,62,63,64,65,66,67,68,69,70,71,72,73,74,75,76,77,78,79,80,83,84,85,86,87,88,89,90,91,92,93,99,100,101,105,106,109,111,112,113,114,115,116,117,118,119,124,125,126,130,131,132,134].

Positive immunotherapy trials should be interpreted according to absolute as well as relative benefit, disease setting, treatment backbone, patient-reported outcomes, cumulative toxicity, generalizability, and treatment burden [9,10,11,14,82,83,84,85,86,87,88,89,90,111,112,124,125,126,137,138,139,140,141,142]. The next phase of gynecologic cancer immunotherapy should therefore address four clinically consequential questions: who truly requires checkpoint blockade; how treatment should be sequenced after prior ICI exposure; what incremental benefit and treatment duration are attributable to ICI within multi-component regimens; and whether prospectively defined resistance mechanisms can guide effective mechanism-matched combinations. Addressing these questions will be more informative than further expansion of unselected checkpoint-based strategies. Ultimately, the value of immunotherapy will be measured not only by response rates or median progression-free survival, but by whether biologically selected strategies safely and meaningfully improve durable outcomes, survivorship, and quality of life.

## Figures and Tables

**Table 1:** Pivotal and practice-informing clinical studies of immunotherapy in gynecologic cancers.

Study	Setting/Design	Regimen	Biomarker or Selection	Key Efficacy Finding	Clinical Interpretation
**Cervical cancer**					
EMPOWER-Cervical 1 [79,80]	Recurrent cervical cancer after platinum-based therapy; randomized phase III	Cemiplimab vs. investigator-choice single-agent chemotherapy	No PD-L1 requirement; squamous and nonsquamous cohorts	Median OS 12.0 vs. 8.5 months; HR for death 0.69	Randomized survival benefit supports PD-1 blockade as a post-platinum option.
KEYNOTE-826 [4,5,6,7]	First-line persistent, recurrent, or metastatic cervical cancer; randomized phase III	Pembrolizumab + platinum/taxane chemotherapy, with or without bevacizumab, vs. placebo + chemotherapy, with or without bevacizumab	Primary testing in PD-L1 CPS ≥ 1, all-comer, and CPS ≥ 10 populations	Final OS in CPS ≥ 1: 28.6 vs. 16.5 months; HR 0.60. All-comer OS: 26.4 vs. 16.8 months; HR 0.63	Defines a first-line pembrolizumab-based chemoimmunotherapy option for PD-L1-positive advanced cervical cancer.
BEATcc [8]	Untreated metastatic, persistent, or recurrent cervical cancer; randomized phase III	Atezolizumab + bevacizumab + platinum/taxane chemotherapy vs. bevacizumab + chemotherapy	All-comer enrollment with PD-L1 testing; not restricted to PD-L1-positive tumors	Median PFS 13.7 vs. 10.4 months; HR 0.62. Interim OS 32.1 vs. 22.8 months; HR 0.68	Supports the complete atezolizumab–bevacizumab–chemotherapy strategy; regional availability and biomarker context remain important.
CALLA [10]	Previously untreated locally advanced cervical cancer; randomized phase III	Durvalumab concurrent with and after chemoradiotherapy vs. placebo + chemoradiotherapy	No biomarker restriction	12-month PFS 76.0% vs. 73.3%; 24-month PFS 65.9% vs. 62.1%; HR 0.84; not statistically significant	Key negative trial showing that immunoradiotherapy benefit cannot be assumed as a class effect.
KEYNOTE-A18 [9,11]	Newly diagnosed high-risk locally advanced cervical cancer; randomized phase III	Pembrolizumab with chemoradiotherapy followed by pembrolizumab maintenance vs. placebo	High-risk locally advanced population; no PD-L1 restriction	3-year OS 82.6% vs. 74.8%; HR for death 0.67; PFS also improved	Moves checkpoint blockade into curative-intent therapy for high-risk locally advanced disease.
COMPASSION-16 [81]	First-line persistent, recurrent, or metastatic cervical cancer; randomized phase III in China	Cadonilimab (PD-1/CTLA-4 bispecific antibody) + platinum-based chemotherapy, with or without bevacizumab, vs. placebo + chemotherapy, with or without bevacizumab	All-comer population; activity reported across PD-L1 subgroups	Median PFS 12.7 vs. 8.1 months; HR 0.62. Median OS not reached vs. 22.8 months; HR 0.64	Practice-informing phase III evidence in the studied setting; comparative value, global applicability, and activity after prior PD-1 exposure remain unresolved.
**Endometrial cancer**					
KEYNOTE-775/Study 309 [13,54]	Previously treated advanced endometrial cancer; randomized phase III	Lenvatinib + pembrolizumab vs. physician-choice chemotherapy	All-comer trial; principal clinical relevance in pMMR/MSS disease	pMMR: median PFS 6.6 vs. 3.8 months; HR 0.60. Median OS 17.4 vs. 12.0 months; HR 0.68	Establishes pembrolizumab–lenvatinib as an evidence-based post-platinum option, especially for pMMR/MSS disease.
LEAP-001/ENGOT-en9 [14]	First-line stage III–IV or recurrent advanced endometrial cancer; randomized phase III	Lenvatinib + pembrolizumab vs. carboplatin–paclitaxel	Primary testing in pMMR and all-comer populations	Did not meet prespecified PFS or OS criteria; pMMR OS HR 1.02 and PFS HR 0.99	Shows that efficacy after platinum failure should not be extrapolated to replace first-line chemotherapy.
NRG-GY018 [15]	Primary advanced or recurrent endometrial cancer; randomized phase III	Pembrolizumab + carboplatin–paclitaxel followed by pembrolizumab maintenance vs. placebo	Prospectively analyzed dMMR and pMMR cohorts	dMMR: 12-month PFS 74% vs. 38%; HR 0.30. pMMR: median PFS 13.1 vs. 8.7 months; HR 0.54	Supports first-line chemoimmunotherapy, with the largest treatment effect in dMMR disease.
RUBY part 1 [16,17]	Primary advanced or recurrent endometrial cancer; randomized phase III	Dostarlimab + carboplatin–paclitaxel followed by dostarlimab maintenance vs. placebo	dMMR/MSI-H subgroup and overall population	dMMR/MSI-H: 24-month PFS 61.4% vs. 15.7%; HR 0.28. Overall OS benefit at second interim analysis: HR 0.69	Reinforces chemoimmunotherapy plus protocol-defined maintenance as an effective first-line framework.
DUO-E [18]	Newly diagnosed advanced or recurrent endometrial cancer; randomized phase III	Durvalumab + carboplatin–paclitaxel followed by durvalumab, with or without olaparib, vs. chemotherapy/placebo maintenance	All-comer with MMR subgroup analyses	ITT PFS improved with durvalumab (HR 0.71) and durvalumab + olaparib (HR 0.55) vs. control	Positive strategy-level result, but the independent contribution of each maintenance component cannot be fully isolated.
AtTEnd [19]	Advanced or recurrent endometrial cancer; randomized phase III	Atezolizumab + carboplatin–paclitaxel followed by atezolizumab maintenance vs. placebo	All-comer and dMMR subgroup analyses	dMMR PFS: not estimable vs. 6.9 months; HR 0.36. Overall PFS: 10.1 vs. 8.9 months; HR 0.74	Further confirms that dMMR tumors derive the most consistent benefit from first-line checkpoint-based therapy.
KEYNOTE-B21/ENGOT-en11 [82]	Newly diagnosed high-risk endometrial cancer after surgery; randomized phase III adjuvant trial	Pembrolizumab or placebo + adjuvant chemotherapy, with or without radiotherapy	All-comer primary analysis; preplanned dMMR subgroup	All-comer DFS not improved; preplanned dMMR subgroup showed a favorable DFS signal (2-year DFS approximately 92% vs. 80% in reports)	Negative in the all-comer population; the dMMR subgroup signal supports further molecularly selected adjuvant investigation but is not independently practice-defining.
**Ovarian cancer**					
JAVELIN Ovarian 100 [83]	Previously untreated stage III–IV epithelial ovarian cancer; randomized phase III	Carboplatin–paclitaxel with avelumab and/or avelumab maintenance vs. chemotherapy alone	Unselected population	Stopped for futility; neither avelumab maintenance nor chemoimmunotherapy improved PFS	Key negative first-line trial; does not support broad avelumab-containing chemoimmunotherapy or maintenance in unselected disease.
JAVELIN Ovarian 200 [84]	Platinum-resistant or platinum-refractory epithelial ovarian cancer; randomized phase III	Avelumab alone or avelumab + pegylated liposomal doxorubicin vs. PLD	Unselected; PD-L1 exploratory	Avelumab + PLD did not significantly improve PFS or OS; PFS 3.7 vs. 3.5 months, HR 0.78	Key negative resistant-disease trial; PD-L1 blockade does not reliably rescue platinum-resistant disease.
ENGOT-ov65/KEYNOTE-B96 [85]	Platinum-resistant recurrent epithelial ovarian, fallopian tube, or primary peritoneal cancer after 1–2 prior systemic regimens; randomized, double-blind phase III	Pembrolizumab + weekly paclitaxel, with or without bevacizumab, vs. placebo + weekly paclitaxel, with or without bevacizumab	PD-L1 CPS assessed; key efficacy population CPS ≥ 1	CPS ≥ 1: median PFS 8.3 vs. 7.2 months; HR 0.72. Median OS 18.2 vs. 14.0 months; HR 0.76	First phase III ICI-based regimen to improve survival in this setting; supports a biomarker- and backbone-specific option rather than broad ICI use in unselected ovarian cancer.
IMagyn050 [86,87]	Newly diagnosed stage III–IV ovarian cancer; randomized phase III	Atezolizumab + bevacizumab + carboplatin–paclitaxel vs. placebo + bevacizumab + chemotherapy	ITT and PD-L1-positive populations	ITT median PFS 19.5 vs. 18.4 months; HR 0.92. PD-L1-positive PFS HR 0.80	Adding atezolizumab to a chemotherapy–bevacizumab backbone did not improve outcomes in an unselected first-line population.
DUO-O [88]	Newly diagnosed advanced ovarian cancer without tumor BRCA mutation; randomized phase III	Chemotherapy + bevacizumab with durvalumab followed by durvalumab + bevacizumab + olaparib vs. standard control	Non-tBRCA population; HRD-positive and ITT primary analyses	Durvalumab + olaparib strategy met PFS endpoints; reported HR 0.63 vs. control	Positive PFS strategy-level result, but the control arm lacked PARP inhibitor maintenance, so the independent PD-L1 contribution cannot be isolated.
ENGOT-OV43/GOG-3036/KEYLYNK-001 [89]	Newly diagnosed advanced BRCA-nonmutated epithelial ovarian cancer; randomized phase III	Chemotherapy with or without pembrolizumab followed by maintenance with olaparib or placebo	BRCA-nonmutated population; subgroup analyses by PD-L1 CPS and HRD	PFS improved with the pembrolizumab–olaparib strategy; OS benefit not established at the reported analysis	Positive PFS result, but absence of an olaparib-alone control prevents attribution of benefit specifically to pembrolizumab.
FIRST/ENGOT-OV44 [90]	Primary advanced ovarian cancer; randomized phase III	Dostarlimab added to platinum-based chemotherapy and niraparib maintenance, with or without bevacizumab, vs. chemotherapy + niraparib-based maintenance	All-comer with subgroup analyses	Median PFS 20.6 vs. 19.2 months; HR 0.85; no OS advantage at reported analysis	Statistically positive but modest PFS improvement; does not support indiscriminate ICI expansion.
**Gestational trophoblastic neoplasia**					
TROPHIMMUN cohorts A/B [91,92]	Chemotherapy-resistant gestational trophoblastic tumors; prospective phase II cohorts	Avelumab monotherapy	Cohort A: low-risk/single-agent-resistant; cohort B: polychemotherapy-resistant; hCG-based response	Cohort A: hCG normalization in 53% of assessable patients. Cohort B: 1 of 7 achieved hCG normalization	Prospective evidence of checkpoint activity, with substantially lower activity after heavier prior chemotherapy; treatment line and disease biology appear important.
CAP 01 [93]	High-risk chemorefractory or relapsed GTN; single-arm phase II	Camrelizumab + apatinib	High-risk refractory/relapsed GTN; hCG response assessment	ORR 55%; complete response/hCG normalization 50%	Supports PD-1 plus anti-angiogenic therapy as a salvage option in selected resistant GTN.

**Evidence interpretation:** Practice-informing studies include randomized trials that define or materially inform current treatment positioning, including important negative phase III trials that delimit non-routine use. Supportive early-phase, retrospective, and rare-tumor studies are summarized separately in Supplementary Table S1. **Abbreviations:** CPS, combined positive score; DFS, disease-free survival; dMMR, mismatch repair-deficient; hCG, human chorionic gonadotropin; HR, hazard ratio; HRD, homologous recombination deficiency; ICI, immune checkpoint inhibitor; ITT, intention-to-treat; MSI-H, microsatellite instability-high; OS, overall survival; PARP, poly(ADP-ribose) polymerase; PD-1, programmed cell death protein 1; PD-L1, programmed death-ligand 1; PFS, progression-free survival; PLD, pegylated liposomal doxorubicin; pMMR/MSS, mismatch repair-proficient/microsatellite stable; tBRCA, tumor BRCA.

**Table 2:** Clinically relevant biomarkers and treatment-selection domains for immunotherapy in gynecologic cancers.

Biomarker or Domain	Clinical Utility	Most Relevant Context	Interpretation for Immunotherapy	Key Caveats and Refs.
dMMR/MSI-H	Validated; clinically actionable	Endometrial cancer; tumor-agnostic use in selected rare gynecologic tumors	Strongest validated enrichment biomarker for PD-1 blockade in endometrial cancer, but not a deterministic predictor of response. Supports PD-1 blockade in recurrent dMMR/MSI-H disease and identifies the subgroup with the largest benefit from first-line chemoimmunotherapy.	Primary resistance and assay discordance occur; integrate MLH1 methylation, histology, molecular class, disease tempo, and prior PD-1 exposure [15,16,17,18,19,20,21,39,40,52,53,109].
PD-L1 expression/CPS	Validated but setting-specific	Persistent, recurrent, or metastatic cervical cancer; PD-L1 CPS ≥ 1 platinum-resistant ovarian, fallopian tube, or primary peritoneal cancer treated with pembrolizumab + weekly paclitaxel, with or without bevacizumab; supportive/investigational use in selected rare tumors	In cervical cancer, PD-L1 CPS ≥ 1 supports pembrolizumab-containing regimens in the appropriate persistent, recurrent, or metastatic setting. In ovarian cancer, CPS ≥ 1 identifies a treatment-selected population within the KEYNOTE-B96 weekly-paclitaxel backbone. Outside these settings, PD-L1 is supportive rather than sufficient for treatment selection.	Spatial/temporal heterogeneity, primary-versus-metastatic sampling, archival tissue, prior therapy, assay clone, scoring method, and inflammatory context affect interpretation. PD-L1 positivity does not guarantee response. KEYNOTE-B96 does not establish PD-L1 as a universal ovarian biomarker [4,5,6,7,29,35,71,72,73,83,84,85,86,87,99,105,106,111,112,118].
POLE pathogenic exonuclease-domain mutation	Prognostic; de-escalation-informing	Endometrial cancer molecular risk stratification and adjuvant de-escalation trials	POLE-mutated tumors are ultramutated, immune-infiltrated, and usually highly favorable. The main clinical value is risk refinement and possible de-escalation, not routine ICI intensification.	Not all POLE variants are pathogenic. Misclassification may cause inappropriate escalation or de-escalation; the added value of immunotherapy in this favorable subgroup remains uncertain [38,41,47,48,49,50,51,55].
TMB-high status	Tumor-agnostic; context-dependent	Selected endometrial cancers, rare gynecologic tumors, and sarcomas when standard options are limited	May support pembrolizumab in selected previously treated unresectable or metastatic solid tumors meeting validated TMB-high criteria. Interpretation is strongest when high TMB reflects true neoantigenic biology.	Numerical cutoffs do not capture neoantigen quality, antigen presentation, HLA context, immune exclusion, or lineage. Do not substitute for MMR, POLE, or molecular classification in endometrial cancer [30,35,96,97,98,119].
HPV-associated antigenicity	Biologic and trial-enabling; not independently predictive	Cervical cancer; HPV-associated vulvar and vaginal cancer; HPV-directed vaccines, TIL therapy, and engineered TCR therapy	Persistent E6/E7 expression provides non-self antigens and supports checkpoint blockade and antigen-directed immunotherapeutic strategies.	HPV positivity is not a complete predictive biomarker. Established tumors may lose antigen presentation, exclude effector cells, or rely on suppressive myeloid/stromal programs [36,37,42,43,44,45,46,79,80,99,100,101,102,103,104,105,106,107,108].
HRD/BRCA and DNA-damage-response context	Treatment-selection for PARP therapy; investigational for ICI prediction	Ovarian cancer maintenance strategies; PARP–ICI trials in ovarian and endometrial cancer	Guides PARP inhibitor use and provides a mechanistic rationale for PARP–ICI combinations through DNA damage, cGAS–STING activation, and type I interferon signaling.	Not a validated stand-alone predictor of checkpoint inhibitor benefit. Trial interpretation is complicated by evolving PARP standards, control arms, and prior PARP exposure [18,22,23,24,25,26,88,89,90,123,124,125,126].

**Interpretive principle:** Routine immunotherapy selection is currently supported most clearly by dMMR/MSI-H, setting-specific PD-L1 CPS, and selected tumor-agnostic TMB-high indications; other domains primarily inform prognosis, combination design, or future trial enrichment. Biological plausibility should therefore not be equated with validated predictive utility. **Abbreviations:** BRCA, BRCA1/2; CPS, combined positive score; dMMR, mismatch repair-deficient; HLA, human leukocyte antigen; HPV, human papillomavirus; HRD, homologous recombination deficiency; ICI, immune checkpoint inhibitor; MSI-H, microsatellite instability-high; PARP, poly(ADP-ribose) polymerase; PD-1/PD-L1, programmed cell death protein 1/programmed death-ligand 1; POLE, DNA polymerase epsilon; TCR, T-cell receptor; TIL, tumor-infiltrating lymphocyte; TMB, tumor mutational burden.

**Table 3:** Rational combination strategies and future priorities for gynecologic cancer immunotherapy.

Strategy	Best Biological/Clinical Fit	What Current Evidence Teaches	Key Unresolved Question
**Chemoimmunotherapy ± maintenance ICI**	Persistent, recurrent, or metastatic cervical cancer; primary advanced or recurrent endometrial cancer; and PD-L1 CPS ≥ 1 platinum-resistant ovarian, fallopian tube, or primary peritoneal cancer treated with weekly paclitaxel ± bevacizumab. Not supported as a broad all-comer strategy in ovarian cancer.	Phase III evidence shows that benefit is specific to tumor lineage, biomarker status, treatment line, and chemotherapy backbone. KEYNOTE-B96 represents a biomarker- and backbone-specific ovarian exception to the negative all-comer JAVELIN Ovarian 100 experience [4,5,6,7,8,15,16,17,18,19,83,85].	Who requires the full chemo-ICI/maintenance strategy, and what is the minimum effective treatment intensity and duration?
**Immunoradiotherapy**	High-risk locally advanced cervical cancer treated with definitive chemoradiotherapy; benefit should not be assumed across all locally advanced populations.	KEYNOTE-A18 provides practice-informing phase III evidence for pembrolizumab with definitive chemoradiotherapy, whereas CALLA was negative with durvalumab, underscoring dependence on regimen, population, and treatment schedule [9,10,11].	Which high-risk patients require ICI with definitive chemoradiotherapy, and for how long?
**Anti-angiogenic therapy plus ICI**	Cervical cancer, pMMR/MSS endometrial cancer, selected resistant GTN, and investigational ovarian settings in which VEGF-driven vascular and immune suppression is clinically relevant.	Practice-informing evidence exists in selected cervical and endometrial settings; evidence in resistant GTN remains investigational. IMagyn050 shows that VEGF inhibition does not automatically create ICI sensitivity in unselected ovarian cancer [3,4,5,6,7,8,13,54,85,86,87,88,93,122,125,126,132].	Can angiogenic–immune biomarkers identify patients in whom VEGF-directed therapy adds clinically meaningful value to ICI?
**PARP inhibitor or DNA-damage combinations with ICI**	Ovarian cancer maintenance and recurrent-disease trials; endometrial maintenance intensification, particularly pMMR/MSS or DNA-damage-response-enriched tumors.	Strong mechanistic rationale is supported by DNA-damage and interferon signaling, but randomized trials provide positive strategy-level signals with unresolved component attribution when controls omit contemporary PARP maintenance or do not isolate the ICI contribution [18,22,23,24,25,26,88,89,90,123,124,125,126].	Does ICI provide independent benefit beyond contemporary PARP-based therapy, and in which biomarker-defined population?
**Dual checkpoint blockade and PD-1/CTLA-4 bispecific antibodies**	Recurrent or metastatic cervical cancer; selected clear-cell gynecologic cancers; biomarker-enriched or post-PD-1 research settings.	Phase III cadonilimab data provide practice-informing evidence for PD-1/CTLA-4 bispecific blockade in the studied cervical cancer setting, whereas nivolumab–ipilimumab remains signal-generating in histology-selected contexts [81,101,114,127,135].	Which patients derive sufficient incremental benefit from dual checkpoint targeting to justify added toxicity?
**HPV-directed vaccines and cellular therapy**	HPV-associated cervical, vulvar, and vaginal cancers, particularly recurrent or metastatic disease after standard therapy or prior ICI.	Therapeutic HPV vaccines, TIL therapy, and HPV E7-directed TCR therapy provide proof of concept, but evidence remains mainly early-phase or nonrandomized [102,103,104,105,106,107].	Can antigen-directed or cellular therapies produce durable post-ICI benefit in prospectively selected HPV-associated disease?
**ADC-based combinations and sequencing**	Recurrent cervical cancer with tissue factor-directed ADCs; FRα-positive platinum-resistant ovarian cancer; and post-platinum, post-ICI, or post-PARP sequencing.	Tisotumab vedotin and mirvetuximab establish the clinical relevance of target-directed cytotoxic delivery, but ADC–ICI synergy remains unproven [12,27,128].	What is the optimal sequence of ADCs and ICI, and does combination therapy add benefit over sequential use?
**Biomarker-guided sequencing, de-escalation, and survivorship**	All tumor types as ICI moves into first-line, maintenance, and curative-intent settings; especially post-ICI cervical/endometrial cancer, ovarian cancer after PARP/bevacizumab, and GTN monitored by hCG.	Resistance and stopping decisions require mechanistic and dynamic biomarkers [30,31,32,33,34,35,94,95,96,97,98,110,120,121]. hCG complements imaging in GTN [75,76,77,78,91,92,93,130,131,132,133,134,136], while iRECIST and irAE guidance support response and toxicity assessment [136,137,138,139,140,141,142,143,144,145].	Can validated dynamic biomarkers safely guide stopping, escalation, or post-ICI treatment selection?

**Interpretive principle:** Positive combination trials establish the value of the tested strategy, but not necessarily the independent contribution of ICI. Clinical interpretation should therefore consider the target population, biomarker context, treatment backbone, control arm, toxicity profile, and post-progression setting. **Abbreviations:** ADC, antibody–drug conjugate; CPS, combined positive score; FRα, folate receptor alpha; GTN, gestational trophoblastic neoplasia; hCG, human chorionic gonadotropin; ICI, immune checkpoint inhibitor; irAE, immune-related adverse event; PARP, poly(ADP-ribose) polymerase; PD-1/PD-L1, programmed cell death protein 1/programmed death-ligand 1; pMMR/MSS, mismatch repair-proficient/microsatellite stable; TCR, T-cell receptor; TIL, tumor-infiltrating lymphocyte; VEGF, vascular endothelial growth factor.

## Data Availability

No new data were created or analyzed in this study. Data sharing is not applicable to this article.

## Supplementary Materials

The following supporting information can be downloaded at: https://www.mdpi.com/article/10.3390/cancers18172771/s1, Table S1. Supportive and emerging clinical studies of immunotherapy in gynecologic cancers. Table S2. Emerging biomarkers, resistance domains, and dynamic monitoring strategies for gynecologic cancer immunotherapy.
