# Supplementary material for: Immunotherapy in Gynecologic Cancers: Current Evidence, Biomarker-Driven Practice, and Future Directions
Source: Cancers (Basel). 2026 Aug 26;18(17):2771. doi: 10.3390/cancers18172771 (PMC13565652; doi:10.3390/cancers18172771)
Supplement: Supplementary file 1 [file cancers-18-02771-s001.zip › Supplementary Table S1.pdf]

Supplementary Table S1. Supportive and emerging clinical studies of immunotherapy in gynecologic cancers

Study	Setting / design	Regimen	Biomarker or selection	Key efficacy finding	Clinical interpretation
**Cervical cancer**					
KEYNOTE-158 [99]	Previously treated advanced cervical cancer; phase II multicohort, nonrandomized	Pembrolizumab monotherapy	PD-L1 CPS assessed; no prospective enrichment for efficacy population	ORR 12.2% overall; all responses occurred in PD-L1-positive tumors (ORR 14.6%); median DOR not reached	Foundational proof of PD-1 activity after chemotherapy; benefit is limited to a minority but can be durable.
**Endometrial cancer**					
KEYNOTE-158 endometrial cohort [53]	Previously treated MSI-H/dMMR advanced or recurrent endometrial cancer; phase II multicohort	Pembrolizumab monotherapy	MSI-H/dMMR required	ORR 48%; median DOR not reached	Supports dMMR/MSI-H as the strongest enrichment biomarker for PD-1 monotherapy in recurrent endometrial cancer.
GARNET [109]	Advanced or recurrent endometrial cancer after platinum; phase I/II expansion	Dostarlimab monotherapy	Separate dMMR/MSI-H and pMMR/MSS cohorts	ORR 43.5% in dMMR/MSI-H and 14.1% in pMMR/MSS disease	Confirms strong enrichment in dMMR/MSI-H disease and limited single-agent activity in pMMR/MSS tumors.
**Ovarian cancer**					
KEYNOTE-100 [111,112]	Recurrent advanced ovarian cancer; phase II	Pembrolizumab monotherapy	PD-L1 CPS explored	ORR 7.4% in cohort A and 9.9% in cohort B; higher CPS associated with numerically higher response	Single-agent PD-1 blockade has limited activity in unselected ovarian cancer.
TOPACIO / KEYNOTE-162 [124]	Recurrent platinum-resistant ovarian carcinoma; single-arm phase I/II	Niraparib + pembrolizumab	No BRCA/HRD restriction; exploratory biomarker analyses	Efficacy-evaluable ORR 18%; disease control 65%; 6- and 12-month PFS rates 31% and 12%	Early PARP–ICI activity signal; randomized validation and better selection are required.
**Gestational trophoblastic neoplasia**					
TROPHAMET [133]	First-line low-risk gestational trophoblastic tumors; phase I/II nonrandomized	Avelumab + methotrexate	FIGO score ≤6; hCG normalization endpoint	Successful hCG normalization 96.2%; no relapses after median 41 months; pregnancy achieved in 13 of 14 patients with pregnancy intention	Promising but investigational earlier-line strategy; comparative validation and careful cost/toxicity assessment are required.
Multicenter retrospective GTN cohort [132]	High-risk chemorefractory or relapsed GTN; multicenter retrospective comparison	Anti-PD-1 therapy + chemotherapy vs anti-PD-1 monotherapy	High-risk refractory/relapsed GTN	ORR 96.8% with combination vs 62.9% with monotherapy; PFS HR 0.06	Suggests greater activity with chemoimmunotherapy, but retrospective design requires prospective confirmation.
**Rare gynecologic malignancies**					
KEYNOTE-158 vulvar SCC cohort [105]	Previously treated advanced vulvar squamous cell carcinoma; phase II cohort	Pembrolizumab monotherapy	PD-L1 assessed; responses occurred regardless of PD-L1 status	ORR 10.9%; median DOR 20.4 months among responders	Durable activity in a minority; disease-specific prospective evidence remains limited.
PEACOCC [113]	Previously treated advanced clear-cell gynecologic cancers; phase II	Pembrolizumab monotherapy	Predominantly MMR-proficient clear-cell tumors	12-week PFS rate 42%; ORR 25%; median OS 14.8 months	Signal of checkpoint activity in predominantly pMMR clear-cell cancers; histology-specific prospective confirmation is needed.
MoST-CIRCUIT [114]	Advanced ovarian and endometrial clear-cell cancers; nonrandomized clinical trial	Nivolumab + ipilimumab	Clear-cell histology	Overall ORR 54%; ORR 55% in ovarian clear-cell and 50% in endometrial clear-cell cohorts	Suggests dual-checkpoint activity in selected clear-cell gynecologic cancers; requires validation.
SARC028 [115,116]	Advanced soft-tissue and bone sarcomas; phase II; not gynecologic-specific	Pembrolizumab monotherapy	Histology-stratified sarcoma cohorts	Soft-tissue sarcoma ORR 18%; no objective responses in the leiomyosarcoma cohort	Cautions against broad extrapolation of checkpoint blockade to uterine sarcomas without biomarker- or histology-specific rationale.

These studies are considered supportive or emerging because they are based predominantly on nonrandomized, early-phase, retrospective, or rare-disease evidence and should not be interpreted as equivalent to established practice-defining evidence.

Abbreviations: CPS, combined positive score; dMMR, mismatch repair-deficient; DOR, duration of response; GTN, gestational trophoblastic neoplasia; hCG, human chorionic gonadotropin; HR, hazard ratio; HRD, homologous recombination deficiency; ICI, immune checkpoint inhibitor; MSI-H, microsatellite instability-high; ORR, objective response rate; OS, overall survival; PARP, poly(ADP-ribose) polymerase; PD-1, programmed cell death protein 1; PFS, progression-free survival; pMMR/MSS, mismatch repair-proficient/microsatellite stable; SCC, squamous cell carcinoma.
