# Supplementary material for: Immunotherapy in Gynecologic Cancers: Current Evidence, Biomarker-Driven Practice, and Future Directions
Source: Cancers (Basel). 2026 Aug 26;18(17):2771. doi: 10.3390/cancers18172771 (PMC13565652; doi:10.3390/cancers18172771)
Supplement: Supplementary file 1 [file cancers-18-02771-s001.zip › Supplementary Table S2.pdf]

Supplementary Table S2. Emerging biomarkers, resistance domains, and dynamic monitoring strategies for gynecologic cancer immunotherapy

Biomarker or domain	Clinical utility	Most relevant context	Interpretation for immunotherapy	Key caveats and refs.
VEGF/angiogenic immune suppression	Combination-design domain	Cervical chemo-ICI–bevacizumab regimens; pembrolizumab–lenvatinib in pMMR/MSS endometrial cancer; ovarian and GTN combinations	Anti-angiogenic therapy may normalize vascular–immune crosstalk, improve immune-cell trafficking, and reduce myeloid-mediated suppression.	No standardized predictive assay. Benefit is regimen- and disease-specific; bleeding, fistula, hypertension, thromboembolism, bowel involvement, and prior pelvic radiation affect use. [3–8,13,54,85–88,93,122,125,126,132]
Ovarian immune architecture: TILs, spatial exclusion, myeloid/stromal programs, and ascites	Emerging; trial-enrichment domain	High-grade serous ovarian cancer and future biomarker-enriched immunotherapy combinations	TILs are prognostic, but response depends on whether effector cells reach tumor nests and overcome suppressive myeloid, stromal, vascular, and ascites-associated programs.	Not standardized for routine care. Sampling region, assay platform, computational thresholds, and clinical cutoffs remain unresolved; best used to enrich mechanistic trials. [56–70,83–87,110–112]
Trophoblastic immune-tolerance markers	Disease-level rationale	GTN, especially chemotherapy-resistant or relapsed disease; selected PSTT/ETT contexts	PD-L1, HLA-G, and maternal–fetal tolerance programs provide a strong biologic rationale for PD-1/PD-L1 blockade in trophoblastic tumors.	PD-L1 may be widely expressed and poorly discriminatory. hCG is highly informative in most GTN but less reliable in PSTT/ETT; prospective selection biomarkers are still needed. [71–78,91–93,130–134,136]
Dynamic monitoring: hCG and ctDNA	Response monitoring; de-escalation research	GTN response assessment; uterine and ovarian cancers; potential cervical cancer post-treatment monitoring	hCG already enables response-adapted management in most GTN. ctDNA may help detect molecular residual disease, early nonresponse, relapse risk, and potentially inform maintenance duration.	ctDNA-guided immunotherapy decisions require prospective validation. Low shedding, timing, assay sensitivity, clonal hematopoiesis, and atypical immune radiographic effects complicate interpretation. [75–78,91–93,120,121,130–134,136]
Antigen presentation, interferon signaling, and HLA context	Resistance biology; investigational	Primary or acquired resistance studies across cervical, endometrial, ovarian, and rare gynecologic tumors	Effective checkpoint blockade requires intact antigen processing, HLA presentation, and interferon responsiveness. Defects may explain resistance despite dMMR/MSI-H, HPV antigenicity, or high TMB.	No routine clinical thresholds in gynecologic oncology. Best used in translational trials, resistance biopsies, and post-immunotherapy sequencing research. [30–35,94–98,110]

Abbreviations: ctDNA, circulating tumor DNA; dMMR, mismatch repair-deficient; ETT, epithelioid trophoblastic tumor; GTN, gestational trophoblastic neoplasia; hCG, human chorionic gonadotropin; HLA, human leukocyte antigen; HPV, human papillomavirus; ICI, immune checkpoint inhibitor; MSI-H, microsatellite instability-high; PD-1/PD-L1, programmed cell death protein 1/programmed death-ligand 1; pMMR/MSS, mismatch repair-proficient/microsatellite stable; PSTT, placental site trophoblastic tumor; TIL, tumor-infiltrating lymphocyte; TMB, tumor mutational burden; VEGF, vascular endothelial growth factor.
